# Genome-Wide Identification, Transcript Profiling and Bioinformatic Analyses of GRAS Transcription Factor Genes in Rice

**DOI:** 10.3389/fpls.2021.777285

**Published:** 2021-11-26

**Authors:** Mouboni Dutta, Anusree Saha, Mazahar Moin, Pulugurtha Bharadwaja Kirti

**Affiliations:** ^1^Department of Plant Sciences, University of Hyderabad, Hyderabad, India; ^2^Department of Biotechnology, Indian Institute of Rice Research, Hyderabad, India; ^3^Agri Biotech Foundation, PJTS Agricultural University Campus, Hyderabad, India

**Keywords:** GRAS genes, rice, stress tolerance, genome-wide analysis, transcription factor, transcript profiling

## Abstract

Our group has previously identified the activation of a GRAS transcription factor (TF) gene in the gain-of-function mutant population developed through activation tagging in rice (in an *indica* rice variety, BPT 5204) that was screened for water use efficiency. This family of GRAS transcription factors has been well known for their diverse roles in gibberellin signaling, light responses, root development, gametogenesis etc. Recent studies indicated their role in biotic and abiotic responses as well. Although this family of TFs received significant attention, not many genes were identified specifically for their roles in mediating stress tolerance in rice. Only *OsGRAS23* (here named as *OsGRAS22*) was reported to code for a TF that induced drought tolerance in rice. In the present study, we have analyzed the expression patterns of rice GRAS TF genes under abiotic (NaCl and ABA treatments) and biotic (leaf samples infected with pathogens, *Xanthomonas oryzae* pv. *oryzae* that causes bacterial leaf blight and *Rhizoctonia solani* that causes sheath blight) stress conditions. In addition, their expression patterns were also analyzed in 13 different developmental stages. We studied their spatio-temporal regulation and correlated them with the *in-silico* studies. Fully annotated genomic sequences available in rice database have enabled us to study the protein properties, ligand interactions, domain analysis and presence of *cis*-regulatory elements through the bioinformatic approach. Most of the genes were induced immediately after the onset of stress particularly in the roots of ABA treated plants. *OsGRAS39* was found to be a highly expressive gene under sheath blight infection and both abiotic stress treatments while *OsGRAS8*, *OsSHR1* and *OsSLR1* were also responsive. Our earlier activation tagging based functional characterization followed by the genome-wide characterization of the GRAS gene family members in the present study clearly show that they are highly appropriate candidate genes for manipulating stress tolerance in rice and other crop plants.

## Introduction

Identification and analysis of transcription factors (TFs) form the essential approaches in Functional Genomics research. TFs bind to DNA or protein sequences and regulate gene expression (Zhang et al., 2018). They play important roles in almost all cellular functions like growth, development, metabolism, signal transduction, resistance/tolerance to abiotic and biotic stress factors, among others. About 320k TFs from 165 different plant species have been reported in literature. Among them, some important transcription factors include WRKY, MBS, MADS, ARF, AP2/EREBP, HB, SBP, bZIP, GRAS and others (Zhang et al., 2018; Shen et al., 2020; Sidhu et al., 2020).

GRAS group of transcription factors are plant specific proteins that were first observed in bacteria and assigned to the Rossman fold methyl transferase superfamily (Zhang et al., 2012). Later, this group radiated towards the ancestors of bryophytes, lycophytes and other higher plants (Cenci and Rouard, 2017; Zhang et al., 2018). A large number of GRAS genes have been identified in various plant species including 34 in Arabidopsis, 60 in rice, 86 in maize, 106 in *Populus trichocarpa* and many others (Tian et al., 2004; Liu and Widmer, 2014; Guo et al., 2017). The higher number of genes in this gene family indicated that the expansion might have happened *via* segmental and tandem duplication events in evolution, followed by their retention (Tian et al., 2004; Huang et al., 2015). Till date, the GRAS family of TFs have been studied in 30 different plant species including Arabidopsis, rice, mustard, lotus, tomato, castor bean, poplar, pine, grapevine and others (Cenci and Rouard, 2017). This gene family has been divided into eight subfamilies in Arabidopsis and rice, while the number varied from eight to 13 in tomato, poplar and castor beans (Tian et al., 2004; Liu and Widmer, 2014; Huang et al., 2015; Xu et al., 2016).

GRAS proteins consist of 400–770 amino acid residues and derive the name from the first three identified members of this family *viz.* Gibberellin-Acid Insensitive (GAI), Repressor of GAI (RGA) and Scarecrow (SCR; Pysh et al., 1999; Bolle, 2004; Zhang et al., 2017). These genes have a conserved C-terminal region, which forms the GRAS domain and a variable N-terminal region. The conserved region or the GRAS domain comprises of five motifs in the following order; Leucine heptad repeat I (LHR I), VHIID motif, Leucine heptad repeat II (LHR II), PFYRE motif and the SAW motif (Pysh et al., 1999). The conserved C-terminal domain is responsible for the transcriptional regulation of the genes that exist under their control. The LHR region is required for protein dimerization and the VHIID is necessary for protein-DNA interactions. PFYRE and SAW are the other important regulatory domains that are present in GRAS TFs. The GRAS genes are mostly nuclear localized except PAT1, which is found in the cytoplasm (Pysh et al., 1999; Tian et al., 2004). The variable N-terminal region consists of intrinsically disordered regions (IDRs,) which are important for molecular recognition during plant development. Due to these IDRs, the GRAS transcription factors are functionally polymorphic (Sun et al., 2012). The members of this gene family integrate environmental and growth regulatory cues and play significant roles in plant development. This family of genes is responsible for a variety of biological functions including gibberellic acid signaling (GAI and RGA of DELLA subfamily and SLR1 of rice; Pysh et al., 1999; Liu and Widmer, 2014; To et al., 2020), SHR and SCR genes for radial root patterning (Helariutta et al., 2000), SCL3 for root elongation (Huang et al., 2015), HAM for shoot meristem formation (Stuurman et al., 2002), PAT genes for phytochrome signaling (Bolle et al., 2000), NSP1 and NSP2 for nodulation signaling pathway (Huang et al., 2015) and some others for abiotic and biotic stress responses (Sun et al., 2012; Zhang et al., 2018; Zeng et al., 2019). In many higher angiosperms, several GRAS genes like *ZmSCL7, AtRGA, AtGAI* were shown to have roles in salt stress tolerance in maize and Arabidopsis (Zeng et al., 2019). *PeSCL7* from *Populus* is associated with the modulation of drought and salt tolerance (Ma et al., 2010). *OsGRAS23* (here named as *OsGRAS22*) was shown to induce drought stress tolerance in rice (Xu et al., 2015). The gene OsGRAS23 (LOC_Os04g50060) as mentioned by Xu et al. (2015) was based on the QTL found in chromosome 4. They followed the nomenclature of Tian et al. (2004). In this study we have considered the recent nomenclature of Liu and Widmer (2014), which is more inclusive and comprehensive. Based on the analysis by Liu and Widmer (2014), OsGRAS23 (as mentioned in Xu et al., 2015) bearing the locus number LOC_Os04g50060 is denoted as OsGRAS22. In the present investigation, OsGRAS23 is a different gene on chromosome 5 bearing the locus id LOC_Os05g31380. Therefore, it has been mentioned now that OsGRAS23 as reported by Xu et al. (2015) is named as OsGRAS22 in this study in order to avoid confusion.

In our previous study (Moin et al., 2016b), we have generated a pool of gain-of-function mutants *via* activation tagging using tetrameric 35S enhancers and screening of some of these mutants for water use efficiency led to the identification of several genes that were associated with the target trait, the water use efficiency. These interesting gain of function mutants included RNA and DNA helicases (*SEN1* and *XPB2*; Dutta et al., 2021), and genes for ribosome biogenesis (*RPL6* and *RPL23A*), protein ubiquitination (*cullin4*) and transcription factors like *WRKY 96* and *GRAS* (LOC_Os03g40080; Moin et al., 2016b). A *GRAS* gene was tagged in the mutant DEB.86 rice line, which showed a high quantum efficiency of 0.82 and a low Δ^13^C value of 18.06‰. Since high photosynthetic efficiency and low carbon isotope ratio are the proxies for high water use efficiency, DEB.86 was further analyzed for other phenotypic characters. This activation tagged line exhibited improved plant height with increased tillering and seed yield and had the *ΨOsGRAS4* gene tagged for activation tagging (Moin et al., 2016b). A total of 60 *GRAS* genes have already been identified in rice (Liu and Widmer, 2014), out of which, *OsGRAS23* has been reported to enhance tolerance to drought (Xu et al., 2015) and *ΨOsGRAS4* has been identified to be associated with enhanced photosynthetic efficiency and water use efficiency with enhanced agronomic features (Moin et al., 2016b). These observations have prompted our group to analyze the GRAS gene family of rice in detail.

In the present study, we have shortlisted 40 genes, one gene representing each paralogous group and provided an experimental basis to identify the potential GRAS genes capable of imparting stress tolerance in rice. We have analyzed the genes selected in the GRAS family for their spatio-temporal and stress induced expression. The phylogenetic relationship among GRAS proteins, their genetic arrangements and structure, *in-silico* analysis of putative promoter elements and protein properties were also studied. This study helps us in the identification of important GRAS genes for stress tolerance, which aids in their further functional characterization.

## Materials and Methods

### Retrieval of GRAS Gene Sequences and Their Nomenclature

Our previous work on gain of function mutants generated through activation tagging technology using the tetrameric 35S elements identified a GRAS gene as a potential player in enhancing water use efficiency in rice (Moin et al., 2016b). Also, Xu et al. (2015) suggested that *OsGRAS23* is involved in inducing drought stress responses in rice. This has led us to undertake literature search in the present study and we observed that Tian et al. (2004) have identified 57 GRAS genes in rice. We searched the accession numbers of all 57 genes in NCBI and performed a BLASTN search in rice genome databases (RGAP-DB- http://rice.uga.edu, Orygenes DB- https://orygenesdb.cirad.fr) and retrieved the locus numbers of 47 genes. Simultaneously, we performed a key word search for GRAS, DELLA, SCR, Monoculm, Chitin- Inducible gibberellin- responsive protein, Gibberellin response modulator protein, Nodulation signaling pathway and Short Root. Subsequently we combined the search results with the 47 genes retrieved from the literature search. Finally, we matched our list of 60 genes with that of Liu and Widmer (2014) and followed the same nomenclature. For more clarity, we had performed a protein database search for the GRAS domain in NCBI, Simple Modular Architecture Research Tool (SMART), Prosite and Pfam databases, and selected 40 genes, one from each of all the paralogous groups for our analyses.

### Genomic Distribution of GRAS Genes

The coordinates of all 60 GRAS genes were obtained from RGAB-DB and were fed in the NCBI Genome Decoration Page. The outputs were combined and the genes were marked for understanding the genomic distribution of *OsGRAS* genes.

### Phylogenetic Relationships of Rice GRAS Genes

In order to understand the evolutionary relationships between the rice GRAS genes, we aligned the amino acid sequences in MEGA7 software followed by the construction of an unrooted phylogenetic tree. The tree was constructed using the Neighbour Joining method with a bootstrap value of 1,000.

### Motif Arrangements and Organization of GRAS Genes in Rice

All the selected 40 GRAS genes were subjected to the MEME suite for conserved motif analysis using default parameters. The number of motif scan was set to 10. Based on the previous investigation of Pysh et al. (1999), the MEME-motifs were further classified into conserved GRAS motifs. The gene organization was studied by subjecting the genomic and coding sequences for analysis in the Gene Structure Display Server (GSDSv2), and the number of exons, introns, untranslated regions (UTRs) etc. were noted.

### *In-silico* Analysis of the Putative Promoter Regions of the Selected GRAS Genes

The *cis*- acting elements in the promoter regions play a major role in the coordinated expression of the genes. Hence, it is crucial to identify these regulatory elements in order to correlate the expression data with the genetic components. We retrieved ≤1kb upstream sequences of all 40 selected GRAS genes under study from the rice genome database and identified important regulatory elements responsible for biotic and abiotic stress responses in them. The identification of these elements was performed by subjecting the sequences in PlantCARE (*Cis*-Acting Regulatory Elements) database and manually mapping them on the chromosomes.

### Biochemical Properties of GRAS Proteins

The sequences of all the 40 shortlisted GRAS genes were subjected to the ExPASyProtParam tool to assess their encoded proteins for amino acid length, molecular weight and theoretical isoelectric points (*p*I). The three-dimensional structures of the proteins and their ligand interactions were studied using 3DLigandSite software (Wass et al., 2010). The structures were then subjected to Phyre2 (Protein Homology/Analogy Recognition Engine v2; Kelley et al., 2015) program for analysis of the protein secondary structure composition. This tool gives an idea of the percentage of secondary structures in a protein, i.e., the percentage of α-helix, β-sheets and the disordered regions in the proteins. The SMART online tool was used to analyse the protein domains and their low complexity regions (LCRs). ExpasyProtParam tool also indicates the GRAVY indices of the proteins, which provide information regarding the hydrophobicity of the subjected proteins. The localization and existence of transmembrane helices in the proteins coded by these genes were predicted using TargetP-2.0 and TMHMM software, respectively.

### Preparation of Plant Material for Studying Gene Expression Under Native and Stress Conditions

For simulated abiotic stress experiments, BPT-5204 (Samba Mahsuri) rice seeds were surface sterilized using 70% ethanol for 50s followed by 4% aqueous sodium hypochlorite solution for 15min and five washes with sterile double distilled water, each of 1min duration. The sterile seeds were germinated on Murashige and Skoog medium for 7d under a 28±2°C for 16h/8h photoperiodic cycle (Bakshi et al., 2017; Saha et al., 2017). The seedlings were then subjected to NaCl (250μm) and ABA (100μm) stress conditions for 60h. The shoot and root samples were collected periodically at 0h, 15min, 3h, 12h, 24h and 60h after exposing the seedlings to the simulated stress treatments. The untreated samples were taken as controls for normalization of gene expression.

For studying the native expression patterns of the GRAS genes, tissue samples from 13 regions in rice seedlings were collected following Moin et al. (2016a) and Saha et al. (2017). These included embryo and endosperm from 16h soaked seeds, plumule and radicle from 3d old germinating seeds, shoot and root tissues from 7d old seedlings and shoot, root, root-shoot transition region, flower, spikes and grain samples from mature 20d old plants post-transfer to the greenhouse. The schematic flowchart for obtaining the results reported in the investigation is provided in Supplementary Figure S1.

In order to study the expression of GRAS genes under biotic stress conditions, leaf samples of 1month old rice plants infected with *Xanthomonas oryzae* pv. *oryzae* [*Xoo* that causes Bacterial Leaf Blight (BLB)] and *Rhizoctonia solani* (that causes Sheath Blight, SB) were taken post 20d and 25d of infection, respectively. Samples from plants of the same age without the pathogen challenge were taken as the controls. The infection protocol was followed as per Saha et al. (2017).

### cDNA Preparation and qRT-PCR

The plant material collected was used to isolate of RNA using Tri-reagent following manufacturer’s protocol (Takara Bio, United Kingdom) and cDNA was prepared using 2μg total RNA samples (Takara Bio, United Kingdom). The cDNA samples were diluted 10 times and an aliquot of 2μl of each sample per reaction was used for quantitative real-time PCR (qRT-PCR). All the primers were designed using Primer3 software and 10μm primer concentration was used per reaction. The PCR program included an initial denaturation step of 94°C for 2min followed by 40cycles of second denaturation of 30s, annealing for 25s and extension at 72°C for 30s. The samples for the current study were taken in biological and technical triplicates, and the fold changes were calculated using the ΔΔC_T_ method (Livak and Schmittgen, 2001). Rice *actin* and *β-tubulin* genes were used as two housekeeping genes for internal normalization. For abiotic and biotic expression studies, housekeeping genes and individual control samples were used for double normalization. In contrast, single normalization was performed using the C_T_ value of housekeeping genes for native expression studies. The graphs were generated using MORPHEUS program and GraphPad Prism software. One way ANOVA was performed using SigmaPlot v.11 software for discerning the significance of statistical differences between samples.

## Results

### Chromosomal Distribution of GRAS Genes in Rice Genome

**Figure 1:**
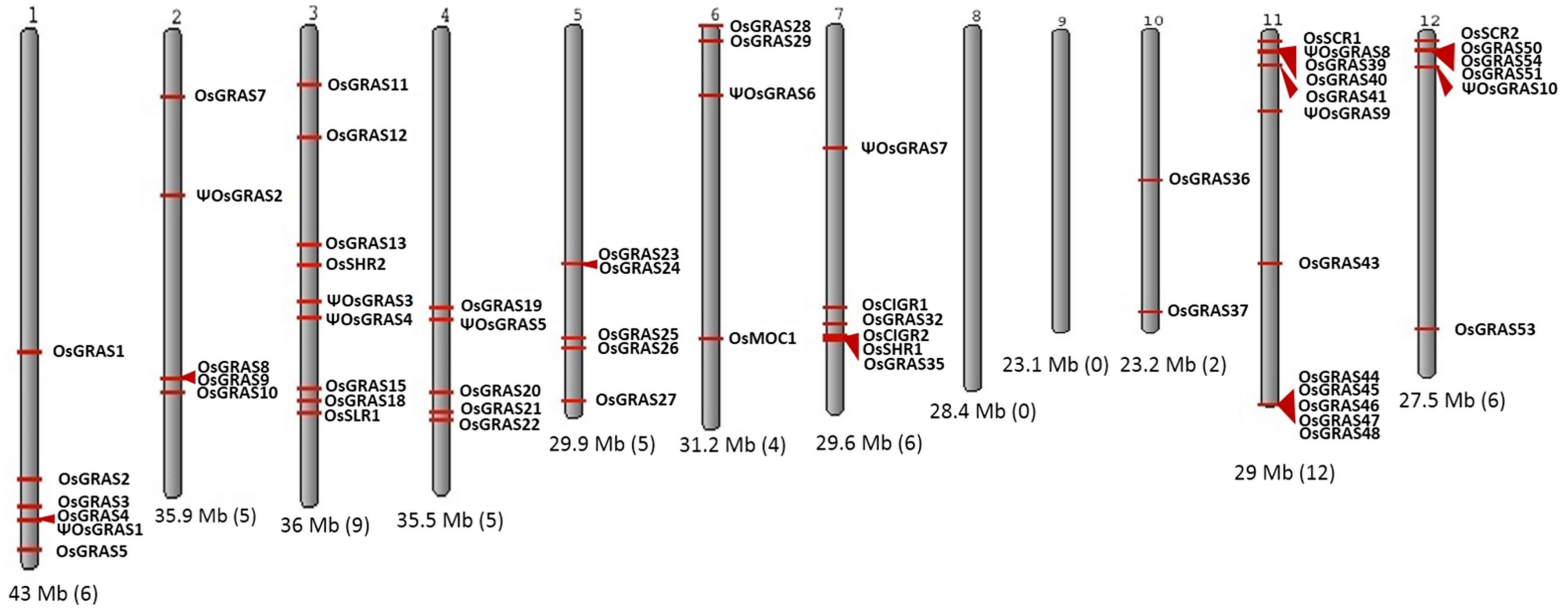
Chromosomal distribution of GRAS genes in rice. Karyotypic representation of rice chromosomes obtained from NCBI Genome Decoration Page. Rice genome carries 60 GRAS genes, which are represented in the figure with red arrows indicating the position of each gene. The size of each chromosome and the number of genes present are provided below each chromosome in brackets.

Liu and Widmer (2014) showed that there are 60 GRAS genes in the genome that are distributed on 10 out of 12 chromosomes of rice. Based on the literature and database search, we observed that chromosomes 8 and 9 did not carry any GRAS genes. The number of genes on a single chromosome ranged from a minimum of two on chromosome 10 to a maximum of 12 on chromosome 11. Among the rest, a total of nine genes were located on chromosome 3, while chromosomes 1, 7 and 12 carried six genes each, chromosomes 2, 4 and 5 exhibited five genes each while the chromosome 6 carried four genes (Figure 1). Out of the 60 genes located, we have shortlisted 40 genes for our study with one representative selected from each of the paralogous groups.

### Analysis of Evolutionary Relationship of *OsGRAS* Genes

**Figure 2:**
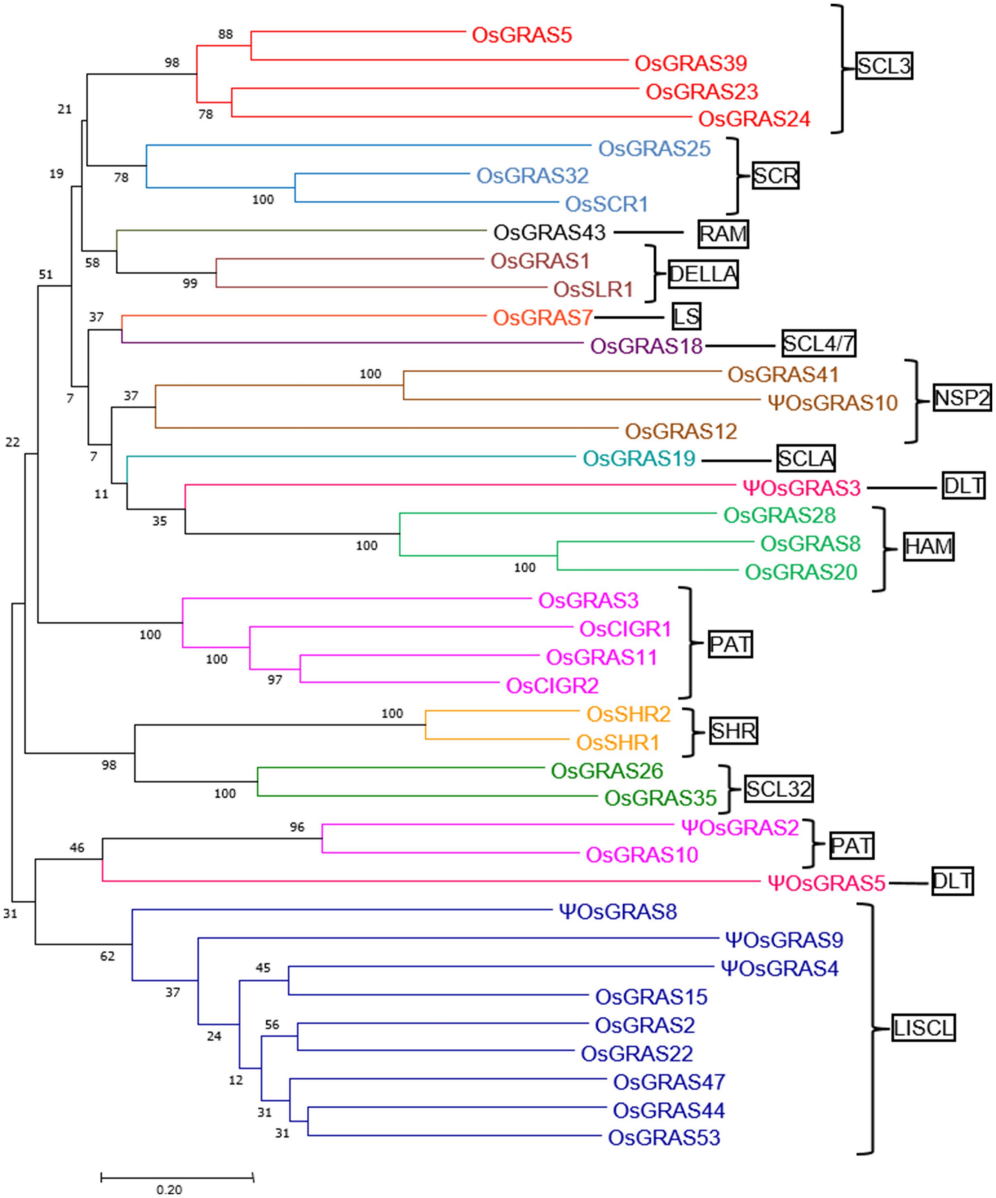
Phylogenetic analysis of *OsGRAS* genes. An unrooted phylogenetic tree showing the evolutionary relationship of *OsGRAS* genes. The tree was constructed using the Neighbour Joining method in MEGA7 software with a bootstrap value of 1,000. The number at each node represents the percentage bootstrap values. Based on the previous literature, the genes have been divided into 14 subfamilies (mentioned in boxes) and each subfamily has been colour coded.

In order to understand the evolutionary relationship among the rice GRAS family of genes, we subjected the retrieved sequences to a phylogenetic analysis (Figure 2) in MEGA7 software. A total of 16 different clusters were observed. These clusters were divided into 14 subfamilies based on a previous report of Cenci and Rouard (2017). Members belonging to the same subfamily were found to cluster together except DLT and PAT subfamilies where some genes belonging to different orthologous groups (according to Cenci and Rouard, 2017) formed separate clusters. Each cluster has been colour coded in the Figure 2. The number of genes found in each subfamily included four in SCL3, three each in SCR, NSP2 and HAM, one in RAM, LS, SCL4/7and SCLA, two in DELLA, DLT, SHR and SCL32, six in PAT and nine in LISCL. LISCL was found to be the largest subfamily with the maximum number of genes getting clustered. *ΨOsGRAS4* and *ΨOsGRAS9* were placed close to LISCL family since these sequences were still unclassified. The highly expressed genes under biotic and abiotic stress conditions belonged to SCL3, SHR, DELLA, HAM and PAT subfamilies.

### Analysis of GRAS Motifs and Gene Organization

**Figure 3:**
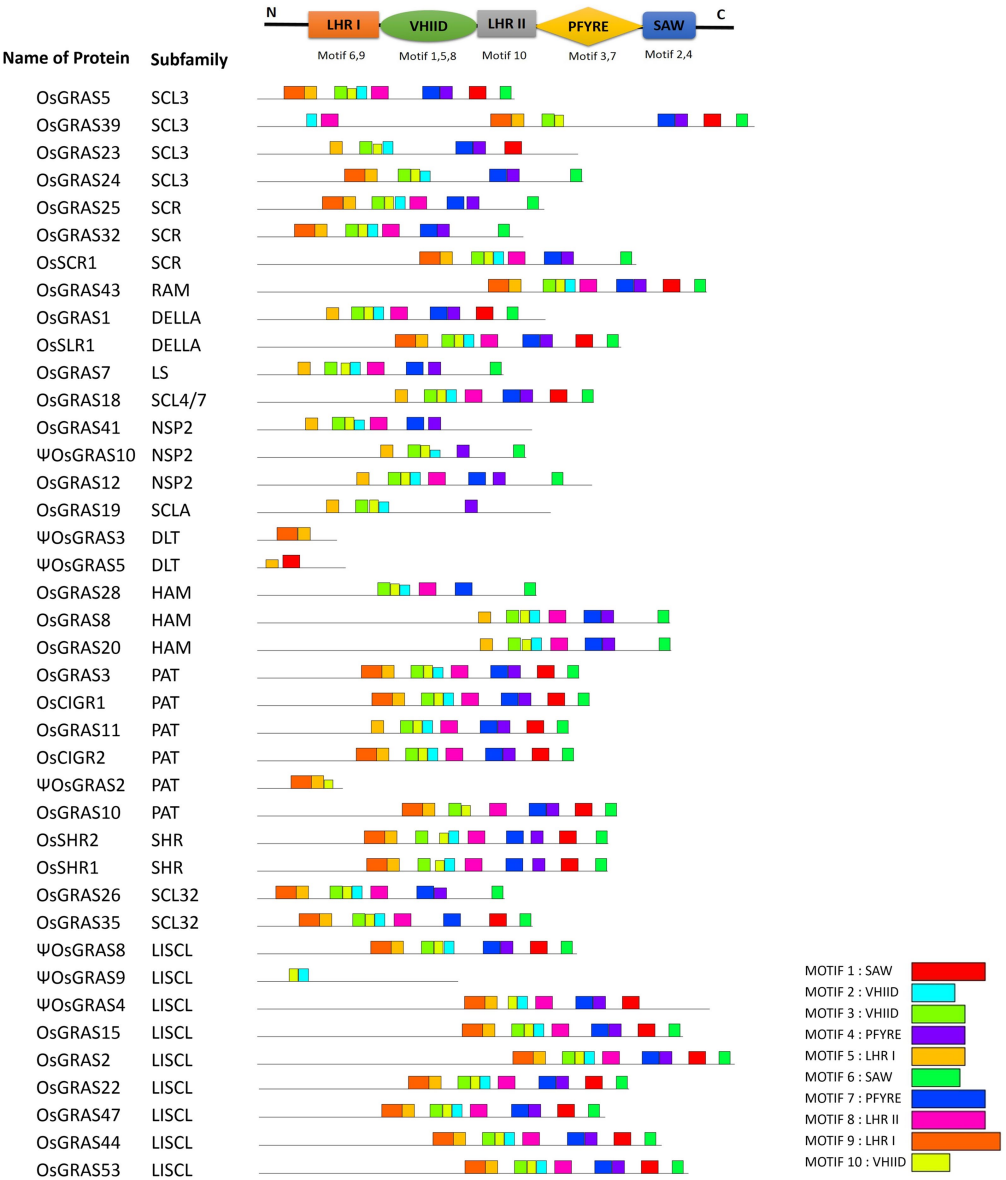
MEME-motif analysis of *OsGRAS* genes. Figure showing the identified MEME-motifs of *OsGRAS* genes. The conserved GRAS-motifs are provided at the top. A search for 10 MEME-motifs was done and each of them has been assigned to the corresponding GRAS-motifs. Each coloured box represents one motif and the legend has been provided below. The genes were organized based on their subfamilies.

The amino acid sequences of selected 40 genes were subjected to MEME analysis for identifying the conserved motifs in rice GRAS gene encoded proteins. A total of 10 motifs were identified, which corresponded to LHR I (motif 5, 9), VHIID (motif 2, 3, 10), LHR II (motif 8), PFYRE (motif 4, 7) and SAW (motif 1, 6) motifs (Figure 3 and Supplementary Figure S2). The C-terminal domain was found to contain the conserved GRAS motifs as reported earlier in literature. However, not all genes exhibited all the 10 MEME-motifs. PAT and LISCL subfamilies carried all the 10 domains, while others like SCR lacked motif 1. Proteins belonging to same subfamily had similar motif composition.

The genomic and cDNA sequences of all the selected genes were subjected to GSDS server to observe the organization of different GRAS genes selected from each of the paralogous groups (Supplementary Figure S3). Based on the map that was generated by the server, it was observed that the genes varied in length and the distribution of exons, introns and UTRs. The majority of genes (31 out of 40 genes studied) lacked introns in their gene structure and were only composed of exonic sequences and UTRs. *OsGRAS11* exon is flanked by a long stretch of UTR at its 5' and 3' ends. It completely lacked introns and is the longest gene in this study (6.7Kb). Ten genes were observed to contain only coding sequences in their structure without any introns and UTRs. Among them, *ΨOsGRAS3* had the smallest sequence of only 414bp. The nine genes carrying introns only in their structure were *OsGRAS3*, *OsGRAS39*, *OsGRAS41*, *OsGRAS43*, *OsSCR1*, *ΨOSGRAS4*, *ΨOsGRAS8*, *ΨOsGRAS9* and *ΨOsGRAS10*. The number of intronic sequences among the genes varied between one (*OsSCR1* and *ΨOsGRAS10*) to a maximum of seven (*ΨOsGRAS4*). All of them showed low (*OsGRAS43*), moderate (*OsGRAS3*, *ΨOSGRAS4* and *ΨOSGRAS8*) and high (*OsGRAS39*, *OsGRAS41*, *OsSCR1*, *ΨOsGRAS9* and *ΨOsGRAS10*) expression levels under abiotic and biotic stress conditions. Six out of nine genes (*OsGRAS41*, *OsGRAS43*, *ΨOSGRAS4*, *ΨOsGRAS8*, *ΨOsGRAS9* and *ΨOsGRAS10*) did not exhibit any UTRs in their structure and were solely composed of introns and exons. The details of genetic organization of rice GRAS genes have been provided in the Supplementary Table S1.

### Putative Promoter Analysis of GRAS Genes and the Search for *cis*-Regulatory Elements

Since diverse expression patterns were observed for different GRAS genes under abiotic and biotic stress conditions, we tried to correlate their expression patterns with the putative regulatory sequences observed in their upstream regions. In order to achieve this correlation, we retrieved 1kb sequences from 5' upstream region of each gene under study from the rice genome database and subjected them to an *in-silico* analysis for the identification of the putative *cis*-regulatory elements observed in them. A total of 18 stress responsive elements were observed in the upstream putative promoter regions of the GRAS genes. These included ABRE or ABA responsive elements, CCAAT box and MYB sites for binding of MYB transcription factors that are reported to be responsive to drought treatments, binding site for MYC transcription factors for defence responses, DRE or dehydration responsive elements, STRE or stress responsive elements, TC-rich repeats for defence and stress responses, and the LTR or low temperature responsive element. Several phytohormones and wound responsive elements were also observed in their upstream regions, which included TCA-element for salicylic acid responses, CGTCA-motif or TGACG-motif as a methyl jasmonate responsive element, GARE-motif, TATC-box and P-box for gibberellin responses, ERE as ethylene responsive elements, TGA-element or AuxRR core or AuxRE for auxin responses, WUN-motif and WRE for responses against wounding, box-S for wounding and pathogen elicitation, and the W-box for binding of WRKY transcription factors.

**Figure 4:**
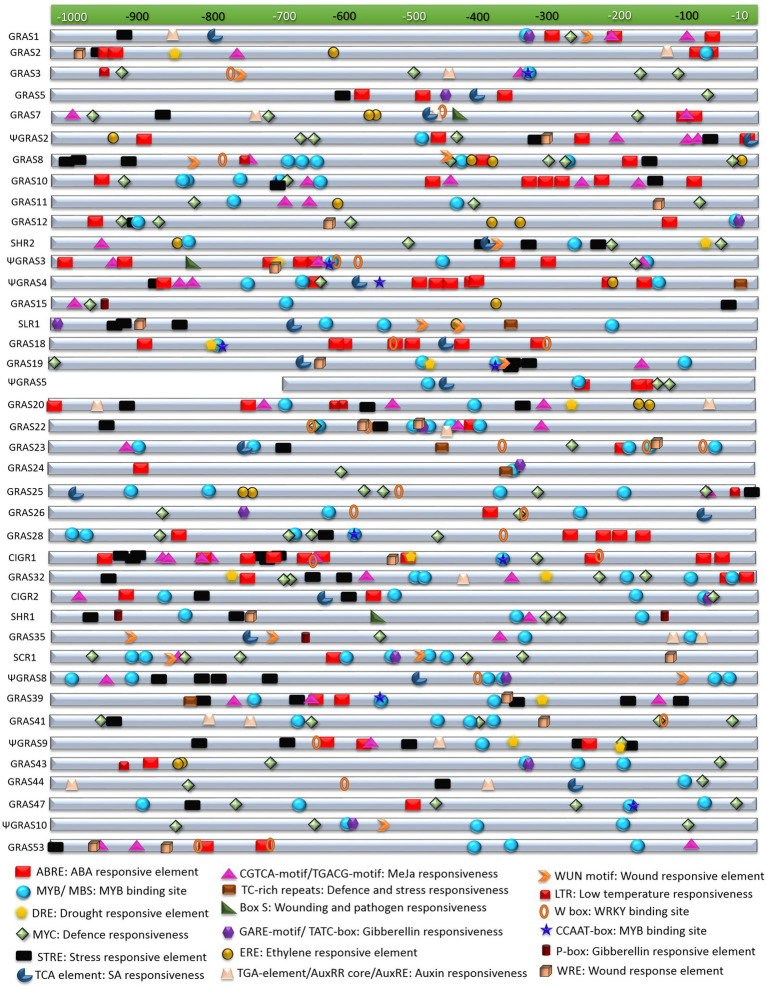
*In-silico* analysis of putative promoter regions of GRAS genes. The selected GRAS genes were subjected to *in silico* analysis for *cis*-regulatory elements in their putative promoter regions (sequence retrieved from about ≤1kb upstream region). This was performed in PlantCARE database and the figure was prepared by mapping the stress regulatory elements in the each of the sequences. The index for each element along with its functions are mentioned below the figure.

*OsGRAS39*, the highly expressive gene under both biotic and abiotic stress conditions in the present study had three copies each of MYB binding factor sites and CGTCA-motif, five copies of STRE, two copies of ABRE and one copy each of DRE, TC-rich repeats and CCAAT-box justifying its expression under different stress treatments. Other responsive genes in both the stresses like *OsGRAS8*, *OsSHR1* and *OsSLR1* had combinations of MYB, STRE, ERE, WUN, TCA, CGTCA and MYC elements in their putative promoter regions. Apart from these, *OsGRAS8* exhibited ABRE, LTR and W-box elements, *OsSHR1* carried a DRE element and *OsSLR1* had copies of TATC, WRE and TC- rich elements. *ΨOsGRAS5*, the only expressive gene in the shoot region had two copies each of MYB and MYC binding elements and three copies of ABRE. Other important abiotic stress responsive genes like *ΨOsGRAS2* and *OsSCR1* were also observed to have multiple copies (upto six) of ABRE, MYB and MYC elements, STRE elements and ERE, CGTCA, GARE and WRE motifs in their 5' upstream regions. *OsCIGR1* that was found to be highly induced under biotic stress conditions carried 10 copies of ABRE, seven copies of STRE, five copies of CGTCA element and one copy each of CCAAT-box, DRE, MYB, MYC and WRE. Other expressive genes under biotic stress conditions included *OsGRAS2*, *ΨOsGRAS3*, *OsGRAS19*, *OsGRAS20* and *OsGRAS23*, which had combinations of TCA-elements, W-box, WRE, ERE, AuxRE, CGTCA-box, box-S and WUN elements apart from other stress responsive elements. The functions of each of these stress responsive elements has been provided in Table 1 and the physical mapping of the important stress responsive elements on the putative promoter regions of the genes is provided in Figure 4.

**Table 1:** List of *cis*-regulatory elements and their functions.

Name of *cis*-element	Function
ABRE	ABA responsive element (Choi et al., 2000)
MYB/MBS	MYB binding site for drought inducibility (Ambawat et al., 2013)
DRE	Dehydration responsive element (Narusaka et al., 2003)
MYC	Transcription factor for stress responses, helps in dehydration induced expression of genes (Tran et al., 2004)
STRE	Stress responsive element (Hwang et al., 2010)
TCA element	Element for salicylic acid responsiveness (Wei et al., 2013)
CGTCA-motif/TGACG-motif	Methyl-Jasmonate responsive element (Wang et al., 2011)
TC-rich motifs	Responsible for defense and stress, transcription regulation (Bernard et al., 2010; Liu et al., 2017)
Box S	Responsive to wounding and pathogen elicitation (Yin et al., 2017); Stress responsiveness (Ding et al., 2019)
GARE-motif/TATC-box	Gibberellin responsive element (Bastian et al., 2010)
ERE	Element for ethylene responses (Oñate-Sánchez and Singh, 2002)
TGA-element/AuxRR core/AuxRE	Element for auxin response (Sakai et al., 1996)
WUN motif	Wound responsive element for biotic stress (Xu et al., 2011)
LTR	Low temperature responsive element (Zhang et al., 2020)
W box	Binding sites for WRKY transcription factors (Dhatterwal et al., 2019)
CCAAT box	Binding site for MYB transcription factors
P-box	Gibberellin responsiveness (Zhang et al., 2020)
WRE	Wound responsive element (Whitbred and Schuler, 2000)

### Properties of GRAS Proteins, Their Ligand Interactions and Domain Analysis

We studied the properties of 40 shortlisted GRAS proteins like amino acid length (aa), molecular weight (kDa) and theoretical *p*I through ExPASyProtParam program. It was observed that the proteins had a molecular weight ranging from 15kDa (ΨOsGRAS3) to 94kDa (OsGRAS39). ΨOsGRAS3 showed a minimum amino acid (aa) length of 137 aa while OsGRAS39 had a maximum length of 854 aa. The *p*I of the proteins ranged from acidic to basic (4.5–10.1) with only eight proteins having a *p*I of more than 7. The majority of the proteins fall under the *p*I range of 4–7. Likewise, the remaining 32 proteins were found to be in the acidic range, i.e., *p*I<7. This is because of the observation that the proteins carried more negatively charged (acidic) amino acid residues like Aspartic and Glutamic acids in their composition as compared to basic amino acid residues. Only OsGRAS39 was found to have an equal number of acidic and basic residues in its composition. According to TargetP-2.0 server, OsGRAS39 was predicted to be localized to the chloroplast while no signal peptides for chloroplast or mitochondria could be specified by the tool for the rest of the proteins.

We have also analyzed the proteins for their three dimensional structures and ligand binding residues in the 3DLigand site and the structures were submitted to the Phyre2 program to analyze their secondary structures like the percentage of disordered regions, α-helix and β-sheets. ΨOsGRAS3 showed a maximum of 71% and OsGRAS8 had a minimum of 31% of α-helical structure. Similarly, maximum (14%) extent of β-sheets were noticed in the secondary structure of OsGRAS32. No β-sheets were present in ΨOsGRAS2 and ΨOsGRAS3. Several metallic and non-metallic ligands were also observed to be interacting with the GRAS proteins, which included Mg^+2^, Ca^+2^, SAM, SAH, NAP, NAD, ATP, Zn^+2^ and Ni^+2^. The three dimensional structures of the proteins along with their interacting ligands have been provided in the Supplementary Figure S4.

Low complexity region are repetitive amino acid sequences found abundantly in the eukaryotic proteins. These play essential roles in protein-protein and protein-nucleic acid interactions (Toll-Riera et al., 2012). It was noted that the number of LCRs in each of the proteins varied from none to a maximum of eight in OsGRAS20 and OsGRAS43, respectively.

Grand average of hydropathicity index or GRAVY index indicates the hydrophobicity of a protein taking into consideration its charge and the size. Usually GRAVY values range from −2 to +2 with more positive values indicating hydrophobicity and more negative values indicating hydrophilicity (Morel et al., 2006). Seven proteins had a positive GRAVY value while the rest 33 proteins had a values lesser than zero, which indicated that the majority of the GRAS proteins are hydrophilic. The list of all the observations have been provided in the Supplementary Table S2.

In order to study the domains present in the genes, we utilized the SMART online tool and observed that all the proteins had at least one GRAS domain with ΨOsGRAS4, ΨOsGRAS8, OsGRAS39, ΨOsGRAS10 exhibiting two GRAS domains. Among them, ΨOsGRAS4 and ΨOsGRAS10 had two internal repeats designated as RPT1 along with two GRAS domains. One DELLA domain and one SCOP domain in addition to the GRAS domain were found in OsSLR1 and OsGRAS18, respectively. DELLA proteins are transcriptional regulators, which function in gibberellic acid signaling by binding with GA receptor, GID1 followed by proteasomal degradation of DELLA domain (Murase et al., 2008). OsGRAS41 had a transmembrane region, OsGRAS43 and OsGRAS53 had two RPT1 domains (internal repeats) along with single GRAS domains. The detailed list of the domains and the LCRs with their sequences have been provided in the Supplementary Table S3. The presence of transmembrane domain in OsGRAS41 was further confirmed through TMHMM software.

### Expression Analysis of GRAS Genes Under Simulated Abiotic Stress Conditions

We have identified a GRAS transcription factor as a potential stress tolerance gene by screening a pool of gain-of-function mutants in rice in our previous study (Moin et al., 2016b). Another report by Xu et al. (2015) suggested the role of *OsGRAS23* (reported as *OsGRAS22* in this study) in drought tolerance in rice. These observations have prompted us to analyze the differential expression pattern of GRAS family of transcription factors under the influence of biotic and simulated abiotic stress conditions in the present study. We have analyzed the expression patterns of 40 selected genes separately in shoot and root tissues at six different time points for two abiotic (NaCl and ABA) and two biotic (BLB and SB) stresses. The native expression patterns of these genes in 13 different tissues were also studied.

Based on the pattern of expression, we have divided the genes as immediate early (IE), early (E) and late (L) responsive genes. Some genes were expressed up to 100 folds after the incidence of stress. Thus, the genes were also categorized as expressive (2–10 fold), moderately expressive (10–30 fold) and highly expressive (≥30 fold) types. Genes showing an upregulation of ≥2 folds were considered as expressive.

**Figure 5:**
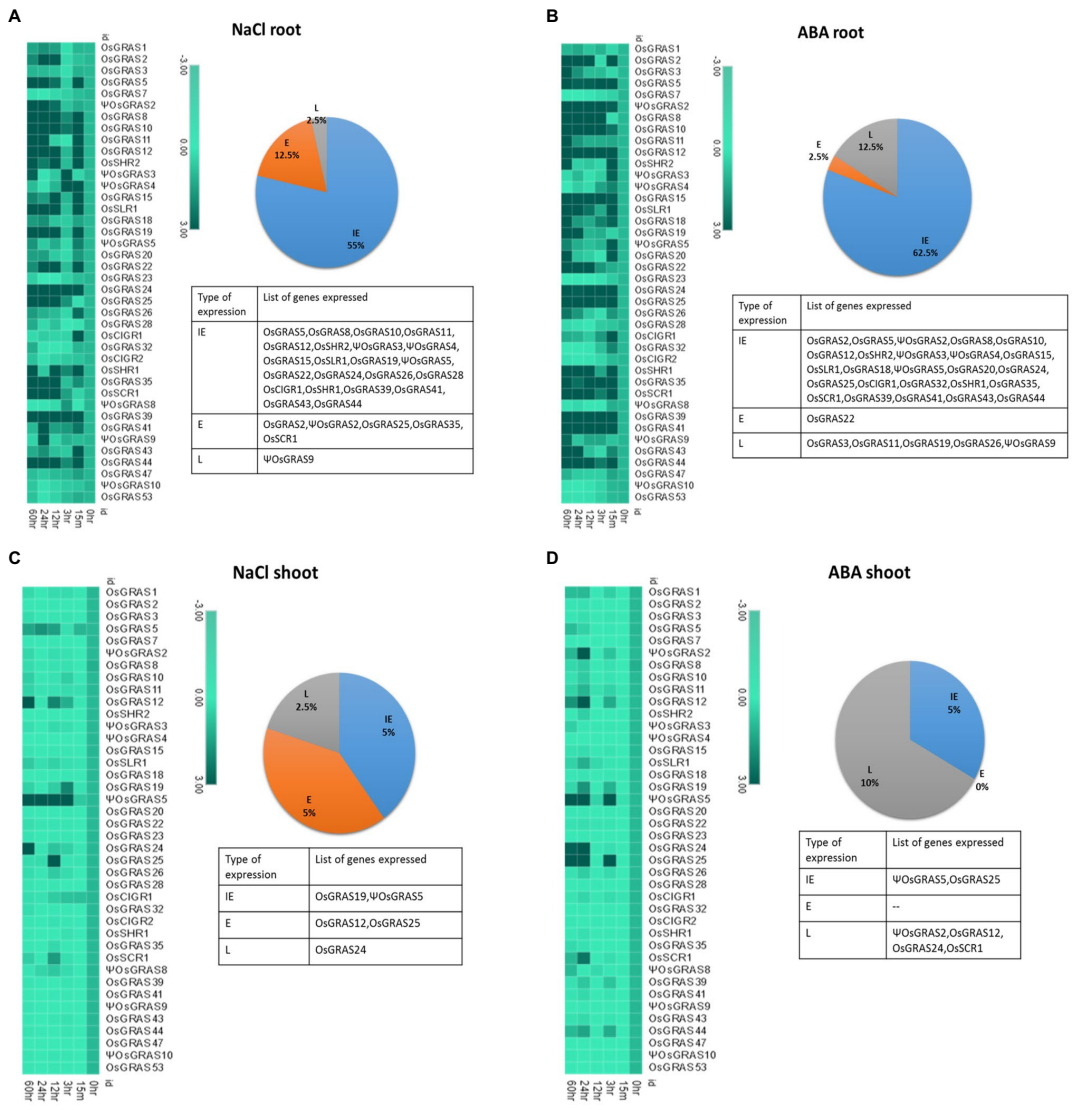
Expression analysis of GRAS genes under abiotic stress. Heat map representation of temporal expression pattern of GRAS genes developed using MORPHEUS program. 7d old seedlings were subjected to NaCl (250μm) and ABA (100μm) treatments and the obtained quantitative real-time values were double normalized using rice *actin* and *tubulin* as the internal reference genes and that of the unstressed samples using the ΔΔC_T_ method. The experiment was conducted separately for root **(A,B)** and shoot **(C,D)** tissues. Percentage of genes upregulated under NaCl and ABA treatments is represented in the form of a pie chart beside their corresponding heat maps. The genes were separated based on their time point(s) of expression and annotated as immediate early (IE), early (E) and late (L) expressive genes. The names of the genes is provided in the list below. The experiment was conducted using biological and technical triplicates (*n*=3), and the mean value was used to plot the heat map in the MORPHEUS software.

The majority of the genes got upregulated in the root (Figures 5A,B) compared to the shoot (Figures 5C,D). As indicated in the pie chart, about 55–60% of the total genes showed IE type expression under both NaCl and ABA treatments. NaCl, however, induced more early (12.5%) responsive genes than the late genes (2.5%) whereas ABA induced more late genes (12.5%) than early ones (2.5%). The list of the expressed genes has been provided in Figure 5. More than half of the IE genes continued their expression till 60h of treatment, while some others became downregulated or showed no expression at all later during the experimental timeline. Under ABA treatment, all highly upregulated genes, i.e., *ΨOsGRAS2*, *OsSHR1*, *OsSCR1* and *OsGRAS39* were IE type and their expression persisted till the last time point of treatment, i.e., 60h. Other IE type genes showed a split before increasing their expression at subsequent time points. Only *OsGRAS39* was highly expressive under both ABA and NaCl treatments (100 fold and 65 fold, respectively). *OsGRAS2*, *ΨOsGRAS2*, *OsGRAS25*, *OsGRAS35* and *OsSCR1* under NaCl and *OsGRAS22* under ABA were early (E) expressed genes respectively, while *ΨOsGRAS9*, *OsGRAS3*, *OsGRAS11* and *OsGRAS26* under ABA and *ΨOsGRAS9* became upregulated under NaCl treatment with late (L) expression.

Twelve and 13 genes (30 and 32%) were mild to moderately expressed, respectively under the ABA treatment, whereas seven genes (17%) were moderately expressive under NaCl treatment and rest 19 genes (47%) exhibited mild expression. Nine genes (22%) under ABA and 13 (32%) genes under NaCl treatment were either downregulated or showed no change in the level of expression. Among them, *OsGRAS7*, *OsGRAS23*, *OsGRAS28* and *ΨOsGRAS8* were downregulated under both the treatments. *ΨOsGRAS3*, *ΨOsGRAS4*, *OsCIGR1* and *OsGRAS32* under ABA treatment and *OsCIGR1* under NaCl treatment showed an immediate expression, but were either downregulated or showed no expression at subsequent time points.

The shoot region did not evidence major GRAS gene expression compared to the root region. However, *ΨOsGRAS5* is the only gene that showed moderate expression (25–30 fold) under both ABA and NaCl treatments in the shoot. This gene was an IE type maintaining its expression till 60h under NaCl, but showed a split before reaching a peak under the ABA treatment. On the contrary, it showed low expression (2–3 fold) in root tissues under ABA and NaCl treatments. Among the other genes that were mildly expressive in both root and shoots were *ΨOsGRAS2*, *OsGRAS12*, *OsGRAS19*, *OsGRAS24*, *OsGRAS25* and *OsSCR1*. The rest of the genes were mainly downregulated or did not show any change in expression in shoot tissues under both the stress treatments. The expression level of all the genes studied has been provided in Supplementary Table S4.

Among the genes studied, some were observed to be expressed only under NaCl or ABA treatments at certain time points, whereas some were found to be expressive under both the treatments. Such overlaps have been depicted in the form of Venn diagrams generated through InteractiVenn software (Heberle et al., 2015) in Supplementary Figure S5. The corresponding list of genes clearly demonstrates that several GRAS genes were up/down-regulated simultaneously under both ABA and NaCl treatments at certain time points. In roots, the expression of 37.5% of the genes (IE type) overlapped under both stress treatments, while *ΨOsGRAS5* (IE) was expressive in shoots only.

### Differential Expression Analysis of *GRAS* Genes Under Biotic Stress

**Figure 6:**
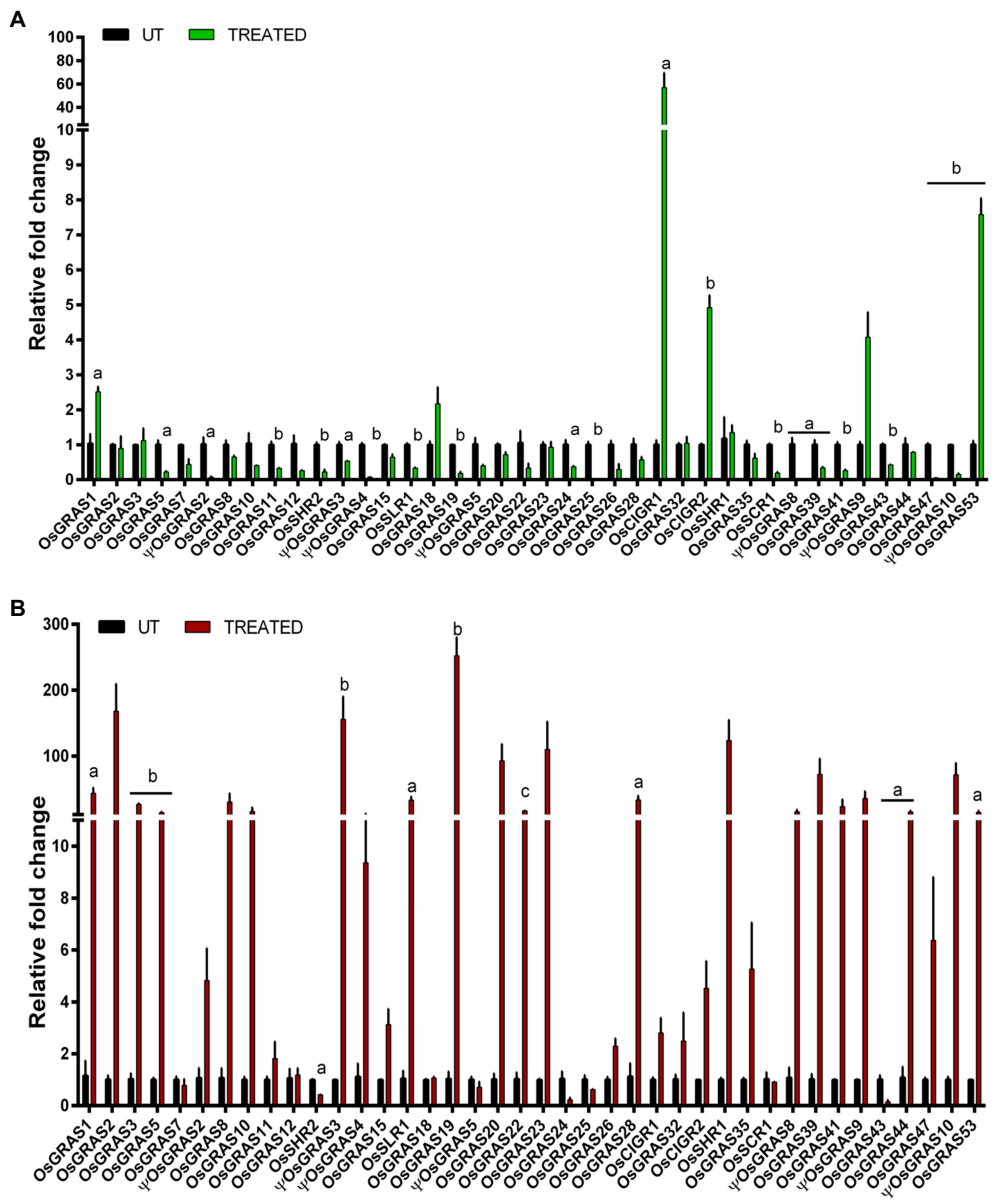
Quantitative real-time expression analysis of GRAS genes under biotic stress. Expression analysis of GRAS genes under the infection of *Xanthomonas oryzae* pv. *oryzae* causing bacterial leaf blight **(A)** and *Rhizoctonia solani* causing sheath blight **(B)** were studied. The genes were double normalized using rice *actin* and *tubulin* as internal reference genes and the C_T_ values of untreated samples by ΔΔC_T_ method. The experiment was conducted using biological and technical triplicates (*n*=3) and a one way ANOVA was performed on the data using SigmaPlot v. 11 to gauge their statistical significane. a represents *p*<0.05, b represents *p*<0.025 and c represents *p*<0.001. The data represent mean±SE.

We have studied the expression of the selected GRAS transcription factor genes in the leaf samples of rice infected with *Xoo* and *R. solani* pathogens that cause BLB and Sheath Blight (SB) diseases, respectively (Figure 6). Six genes were upregulated in BLB of which five (*OsGRAS1*, *OsGRAS18*, *OsCIGR2*, *ΨOsGRAS9*, *OsGRAS53*) showed low expression while one gene (*OsCIGR1*) was highly upregulated up to 57 folds. More genes were upregulated in SB infected leaves compared to the BLB treated ones. Out of the 30 expressed genes in SB infected leaves, only 12 showed high expression levels while the rest of the genes exhibited low to moderate expression. *OsGRAS2*, *ΨOsGRAS3*, *OsGRAS19*, *OsGRAS20*, *OsGRAS23* and *OsSHR1* were expressed by ≥100 folds under the SB treatment. A total of 22 genes in BLB and three in SB treated samples were downregulated. Those that were downregulated in SB treated samples (*OsSHR2*, *OsGRAS24* and *OsGRAS43*) were also downregulated in BLB treated leaves. Twelve genes under BLB and seven under SB did not show any changes in their expression levels.

### Native Expression Analysis of *GRAS* Genes in Various Tissues at Specific Developmental Stages in Rice

In order to study the native expression patterns of GRAS transcription factors in different tissues of the rice plant, we performed qRT-PCR analysis of 13 different tissues, which included shoot, root, root-shoot transition, flag leaves, flower, spikes and grain of mature

**Figure 7:**
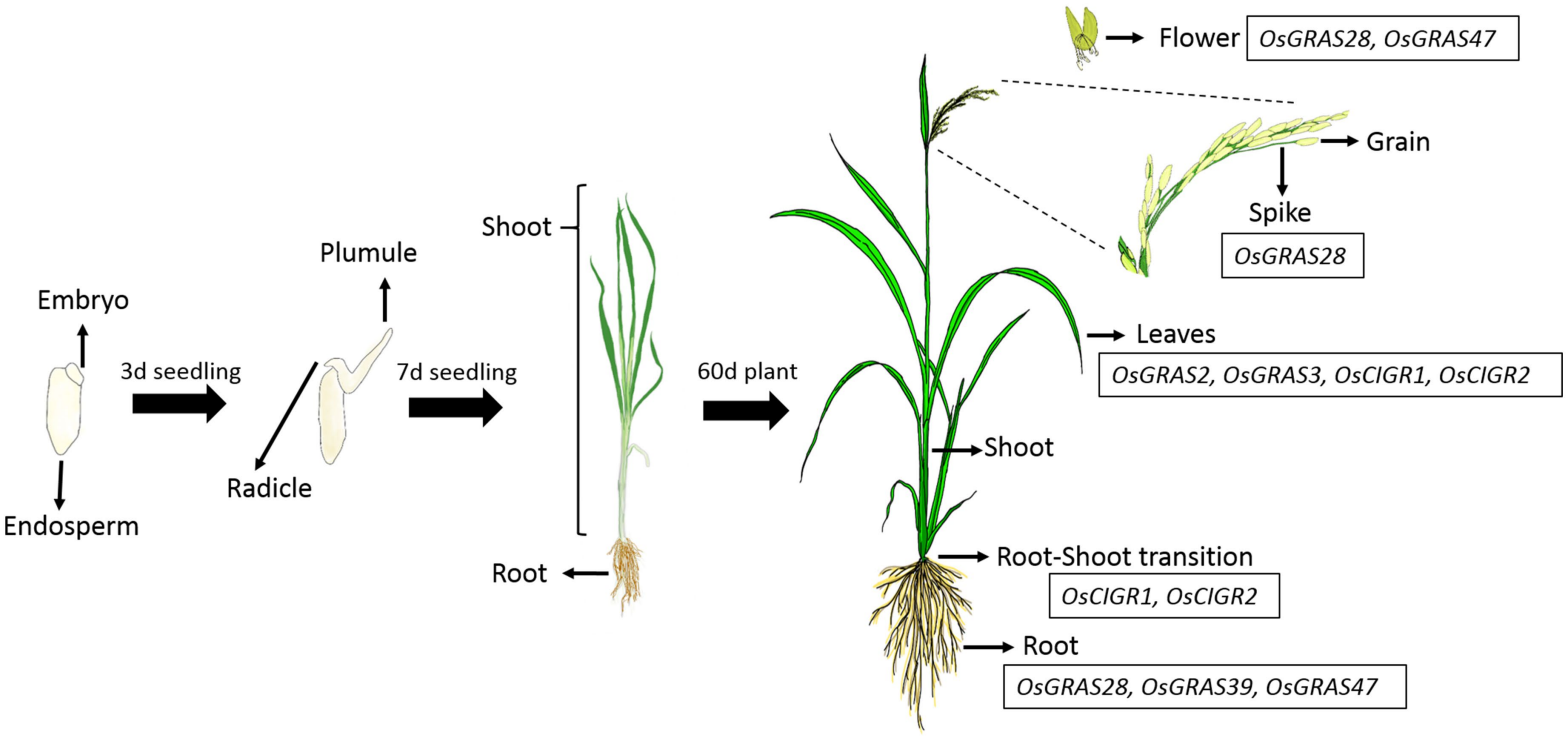
Spatial regulation of OsGRAS genes. The native expression pattern of GRAS genes was studied in 13 different developmental tissues of rice plant. Majority of the genes were downregulated with some of them getting upregulated in mature vegetative and reproductive tissues. The list of the genes expressed in each tissue is mentioned in the boxes beside them. The figure has been adopted from Saha et al. (2017).

**Figure 8:**
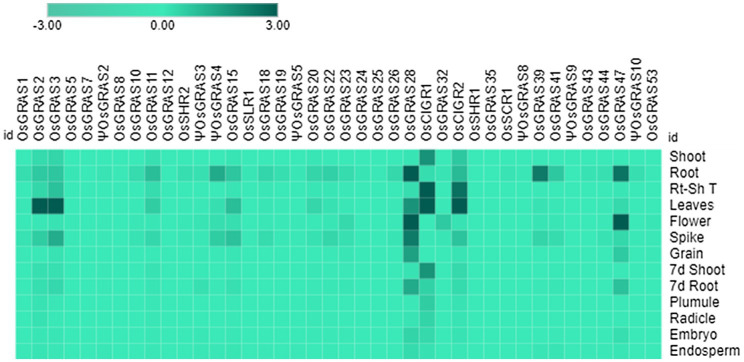
Native expression analysis of GRAS genes. Heat map representing the spatial expression pattern of GRAS genes under 13 different developmental stages of rice. The experiment was conducted using biological and technical triplicates (*n*=3) and the mean expression values were used to generate the map using the MORPHEUS program. The data was single normalized using rice *actin* as the internal reference gene.

20d old plants (after shifting to the greenhouse), shoot and root of 7d old seedlings, 3d old plumule and radicle, embryo and endosperm of 16h germinating seeds (Figure 7). The mean values were used to plot the heat maps (Figure 8).

The expression analysis showed a conspicuous downregulation of all genes in most of the tissues particularly in plumule, radicle, embryo and the endosperm. Out of 40 selected genes, only seven were expressed in mature vegetative and reproductive tissues. *OsGRAS2* and *OsGRAS3* were upregulated only in mature leaves, *OsGRAS28* in 20d root, flower and spike, *OsCIGR1* and *OsCIGR2* in root-shoot transition and leaves, *OsGRAS39* in 20d root and *OsGRAS47* in 20d root and flower. Out of these seven genes, five were upregulated either in the roots or in the root-shoot transition region indicating the preference of GRAS genes towards expression in the root tissue. This is also in accordance with the expression analysis under abiotic stress condition, where the genes were highly expressive in roots rather than in the shoot tissue. Three out of seven mildly expressive genes were upregulated in flower and spike of 20d old plants while none of them expressed in the grain. *OsGRAS39* that was upregulated in root tissues under native conditions is highly expressive in roots under abiotic stress conditions also responding immediately after the application of stress treatment. This might be an indication of its tissue specificity and its potential as a stress tolerance transcription factor gene for genetic manipulation in rice.

## Discussion

Being sessile, plants cannot escape the onslaught from environmental stresses like cold, heat, drought etc., nor can they avoid harmful interactions with microorganisms like fungi and bacteria (Lin et al., 2017). Such adversities impose a threat to agricultural productivity and sustainability. In order to support the burgeoning global population, the development of stress tolerant crops is of utmost importance. Characterization of insertional mutants is an important functional genomics based method of identifying novel genes responsible for inducing stress tolerance in crop plants (Cushman and Bohnert, 2000). TF genes are of particular importance in this context as they act upstream in the pathway(s) and control the expression of several genes working under their control. Because of this, the manipulation undertaken using TF genes as ‘Master’ genes would render the plant more accommodative towards the particular stress under consideration. Our previous studies have identified several key players for stress tolerance in an *indica* rice variety *via* enhancer based activation tagging method and a GRAS transcription factor gene, *ΨOsGRAS4* was one of the important genes that was identified in the study along with others for enhanced water use efficiency associated with enhanced photosynthetic efficiency (Moin et al., 2016b).

### Evolutionary Relationships, Gene Organization and Protein Properties of Rice GRAS Genes

Plants use certain acclimation and adaptive measures to cope up with the impending stress, which is mostly modulated through the action of hormones and regulator genes (Lin et al., 2017). Thus, understanding the expression patterns of GRAS family of genes, which play a key role in gibberellin signaling and their spatio-temporal regulation help us identify the candidate genes for improving the endogenous defence ability of plants, particularly rice in the present context. In this study, we have shortlisted important GRAS genes responsible for abiotic and biotic stress tolerance. We have also studied the *in-silico* properties of these genes and have correlated them with our expression data.

According to the published evidence that is available (Liu and Widmer, 2014), 60 GRAS genes were reported in the rice genome, which are distributed on all the 12 chromosomes except chromosome numbers 8 and 9. Highest number of GRAS gene density was observed on chromosome 11. We have selected 40 genes for our study, drawing one member representing each paralogous group. The availability of high quality genomic sequences enabled us to get an insight into the phylogenetic, genomic and protein properties of the GRAS genes in rice. In our analysis, we have classified the genes into 14 subfamilies (Cenci and Rouard, 2017) of which LISCL constituted the maximum number of genes. However, most expressive genes belonged to SCL3, SHR1, DELLA, HAM and PAT subfamilies.

The 10 MEME-identified motifs were categorized into five conserved C-terminal GRAS motifs. Genes belonging to the same subfamily exhibited similar motif arrangements, but this varied within the subfamilies, which might be due to the diverse biological functions of GRAS genes. This group of proteins were reported to have originated in bacteria, which later expanded into eukaryotic genomes *via* horizontal gene transfer and repeated duplication events with the possible retroposition of intronless genes (Huang et al., 2015). Our genomic organization study revealed 31 *OsGRAS* genes out of 40 selected, to be intronless and this observation is in line with previous studies. Several interacting metallic and non-metallic ligands associated with GRAS genes along with their hydrophilic nature (as indicated by the GRAVY index) indicated their involvement in cell signaling, catalysis and protein-protein interactions (Ulucan et al., 2014; Jing et al., 2017).

The majority of the genes were observed to have a *p*I less than seven and were found to be rich in negatively charged amino acids like Glutamic acid and Aspartic acid. This makes the interactions of GRAS proteins very specific as proteins with low *p*I tend to minimize the chances of non-specific interactions with nucleic acids and other acidic proteins (Takakura et al., 2015). All GRAS genes have at least one GRAS domain, but some were found to have two or possess a DELLA domain, which is known to have important role in gibberellic acid signaling (Urbanova and Leubner-Metzger, 2018).

### Differential Expression Patterns of *OsGRAS* Genes and Their Spatio-Temporal Regulation

Based on external cues, spatio-temporal regulation of gene transcription is required to control the concentration of particular transcripts and proteins in the cells for their adjustment to the environmental changes. Most of the GRAS genes were observed to be upregulated in roots with 55–60% of them showing IE type of gene expression. In plants, stress responses can be divided broadly into early and late response types. Early responsive genes are expressed within minutes of stress induction and this provides protection and repair from the initial stress. Such response “alarms” the plant to prepare for further stress tolerance or avoidance. On the other hand, late responsive genes are mostly involved in protein synthesis that regulates downstream genes, thereby responding to the “adaptation” part of stress mediation (Bahrami and Drabløs, 2016; Lin et al., 2017). *ΨOsGRAS9* was observed to be late expressive under both treatments in root indicating that it might have an important role in subsequent steps of stress amelioration. Interestingly, 50–60% of IE expressed genes continued to express till the end point of the treatment. Among them, *OsGRAS39* was the only gene that was observed to be highly expressive under both NaCl and ABA treatments in roots. Apart from this, *ΨOsGRAS2*, *OsSHR1* and *OsSCR1* continued their expression till 60h under ABA treatment. This probably indicates that these constitute an important set of genes required by the plant throughout for stress remediation. Others like *ΨOsGRAS3*, *ΨOsGRAS4*, *OsCIGR1* and *OsGRAS32* under ABA and *OsCIGR1* under NaCl were the genes induced initially (IE type), which either stopped expressing or got downregulated at subsequent time points. These genes are probably required for initial stress responses whose function is later on taken up by the other downstream genes in the signaling cascade. *ΨOsGRAS5* (IE type) was found to be the only moderately expressive gene in shoot under both stress conditions. Since root is the first organ to perceive the stress signal, it induces a signaling cascade that extends towards the shoot. Such preferential expression of *GRAS* genes in roots over shoots indicates their important role in stress responses (Janiak et al., 2016). Also, previous studies indicated that it is probable that the role of these genes in pattern formation and signal transduction enables them to be more expressive in roots (Pysh et al., 1999).

Rice productivity is severely hampered by BLB and SB diseases. BLB infection during tillering stage can cause a yield decline of 20–40%, while it can reduce crop productivity by 50% at a younger stage. Upto 45% yield losses in rice are caused by SB infections (Chukwu et al., 2019; Singh et al., 2019). Thus, identification of key genes and understanding their expression patterns are important for developing tolerant varieties of rice for these diseases. Under BLB treatment, only six genes (15%) were expressive compared to 30 (75%) expressive genes under SB infection. Quite a number of genes were expressive under SB infection and both abiotic stress conditions. Noteworthy among them are *OsGRAS39*, *OsGRAS8*, *OsSHR1* and *OsSLR1*. Thus, these genes can be considered to be quite important as they are expressive under both stress conditions with important roles in disease resistance. Majority of the genes were highly expressive in ABA treated roots and SB infection. The presence of multiple stress responsive elements in their putative promoter regions are corroborated by our expression data and these observations indicate their probable roles in improving plant defence against biotic and abiotic stress.

Out of 13 different developmental tissues, only seven genes were induced in mature vegetative and reproductive tissues. Proteins belonging to DELLA subfamily of GRAS transcription factors are known to be negative regulators of seed germination as bioactive gibberellic acid causes proteasomal degradation of such proteins for gibberellin signaling to occur (Urbanova and Leubner-Metzger, 2018). This explains the downregulation of all genes in plumule, radicle, embryo and endosperm. Thus, *OsGRAS* genes might be associated with developmental regulation in mature rice plants. The induction of *OsGRAS28* belonging to HAM subfamily and *OsGRAS47* belonging to LISCL subfamily in reproductive tissues indicates their role in floral development. This can be correlated with the expression of *PhHAM* genes of *Petunia hybrida* (Stuurman et al., 2002), *AtSCL6* and *AtSCL27* genes of *Arabidopsis* (Fan et al., 2017) and *PtGRAS67* of *Populus* (Liu and Widmer, 2014) flowers. All these genes belonging to HAM subfamily were reported to be involved in floral differentiation. LISCL subfamily gene from lily plants were also reported to be associated with miscrosporogenesis (Bolle, 2004).

GRAS proteins are involved in growth signaling and plant developmental regulation. *OsGRAS39* was expressive in roots under native as well as abiotic stress conditions indicating its tissue specificity. This gene belongs to SCL3 subfamily, which is known for modulating GA signaling in roots *via* protein–protein interactions (Weng et al., 2020). This group of proteins are involved in maintaining gibberellic acid homeostasis in roots, which in turn help them in controlling root development (Heo et al., 2011). During salt or ABA stress, roots are the primary organs to perceive the stress signals and initiate a signaling cascade towards the shoot. Therefore, *OsGRAS39* (belonging to subfamily SCL3) along with other notable candidate genes like *OsSHR1* (belonging to SHR subfamily) and *OsSLR1* (belonging to SCR subfamily) might have an impact on the SCL3-DELLA interaction and SHR/SCR pathway in the root tissue leading to stress tolerance. Hence, high expression pattern of the above mentioned genes can be further exploited for their potential role in stress tolerance. A schematic diagram has been provided in the Supplementary Figure S6.

GRAS gene family has been studied extensively in many plant species, but we have successfully provided a backdrop based on which future exploration on rice GRAS genes can be done. The differential expression patterns of these genes indicates their importance in stress remediation. Our study provides an insight into the role of GRAS genes in stress tolerance along with their spatio-temporal regulation. Based on this report, it would be possible to pick up important genes that can be further manipulated for developing stress tolerant varieties in rice and other related crops.

## Data Availability Statement

The original contributions presented in the study are included in the article/Supplementary Material, further inquiries can be directed to the corresponding authors.

## Author Contributions

PK and MD designed the experiments and prepared the manuscript. MD performed the experiments. AS helped in the qRT-PCR experiments and analysis. MM identified GRAS gene from activation tagged lines and conceived the initial idea. PK supervised the work. All authors contributed to the article and approved the submitted version.

## Conflict of Interest

The authors declare that the research was conducted in the absence of any commercial or financial relationships that could be construed as a potential conflict of interest.

## Publisher’s Note

All claims expressed in this article are solely those of the authors and do not necessarily represent those of their affiliated organizations, or those of the publisher, the editors and the reviewers. Any product that may be evaluated in this article, or claim that may be made by its manufacturer, is not guaranteed or endorsed by the publisher.
