# Supplementary material for: Genome-Wide Identification, Transcript Profiling and Bioinformatic Analyses of GRAS Transcription Factor Genes in Rice
Source: Front Plant Sci. 2021 Nov 26;12:777285. doi: 10.3389/fpls.2021.777285 (PMC8660974; doi:10.3389/fpls.2021.777285)
Supplement: Supplementary file 1 [file Data_Sheet_1.pdf]

For abiotic expression analysis

- Around 550 rice seeds (BPT-5204) were surface sterilized and germinated on Murashige and Skoog media for 7 days.

- After 7 days healthy seedlings of similar size were grouped separately. Each set comprised of 25 seedlings, which were subjected to NaCl and ABA stress treatments at different time points along with the control (as mentioned in the Materials and Methods section 2.7).

- For each time point per treatment, 5 seedlings were collected. Shoot and root samples were stored separately for the subsequent expression analysis.

- Sample collection was followed by RNA isolation, cDNA preparation and the subsequent qRT-PCR analysis (detailed protocol is mentioned in Materials and Methods section 2.8).

- The list of the important genes expressed during the simulated stress treatments is provided in the tables below:

NaCl Root	
Type of expression	List of genes expressed
IE	OsGRAS5,OsGRAS8,OsGRAS10,OsGRAS11, OsGRAS12,OsSHR2,ΨOsGRAS3,ΨOsGRAS4, OsGRAS15,OsSLR1,OsGRAS19,ΨOsGRAS5, OsGRAS22,OsGRAS24,OsGRAS26,OsGRAS28 OsCIGR1,OsSHR1,OsGRAS39,OsGRAS41, OsGRAS43,OsGRAS44
E	OsGRAS2,ΨOsGRAS2,OsGRAS25,OsGRAS35, OsSCR1
L	ΨOsGRAS9

ABA Root	
Type of expression	List of genes expressed
IE	OsGRAS2,OsGRAS5,ΨOsGRAS2,OsGRAS8,OsGRAS10, OsGRAS12,OsSHR2,ΨOsGRAS3,ΨOsGRAS4,OsGRAS15,OsS LR1,OsGRAS18,ΨOsGRAS5,OsGRAS20,OsGRAS24, OsGRAS25,OsCIGR1,OsGRAS32,OsSHR1,OsGRAS35, OsSCR1,OsGRAS39,OsGRAS41,OsGRAS43,OsGRAS44
E	OsGRAS22
L	OsGRAS3,OsGRAS11,OsGRAS19,OsGRAS26,ΨOsGRAS9

NaCl Shoot	
Type of expression	List of genes expressed
IE	OsGRAS19,ΨOsGRAS5
E	OsGRAS12,OsGRAS25
L	OsGRAS24

ABA Shoot	
Type of expression	List of genes expressed
IE	ΨOsGRAS5,OsGRAS25
E	--
L	ΨOsGRAS2,OsGRAS12, OsGRAS24,OsSCR1

For native expression analysis

- For samples of embryo and endosperm, 100 seeds were soaked in tissue paper for 16h. Post 16h, the seeds were segregated into three sets, each containing 20 seeds, and the samples were collected.

- Around 200 seeds were surface sterilized and allowed to grow on MS media to obtain the samples for rest of the tissues. After 3 days of germination, plumule and radicle samples were collected from 20 seedlings. As mentioned in the Materials and Methods Section 2.7, samples were collected separately from 7 days old seedlings and from mature plants post greenhouse transfer. Samples from 5 plants and seedlings were pooled during collection.

- Sample collection was followed by RNA isolation, cDNA preparation and the subsequent qRT-PCR analysis (detailed protocol is mentioned in Materials and Methods section 2.8). The list of expressed genes is provided in the Figure 7 of the manuscript.

Supplementary Figure S1: Schematic flowchart showing the methodology for obtaining the results during abiotic and native expression analyses.

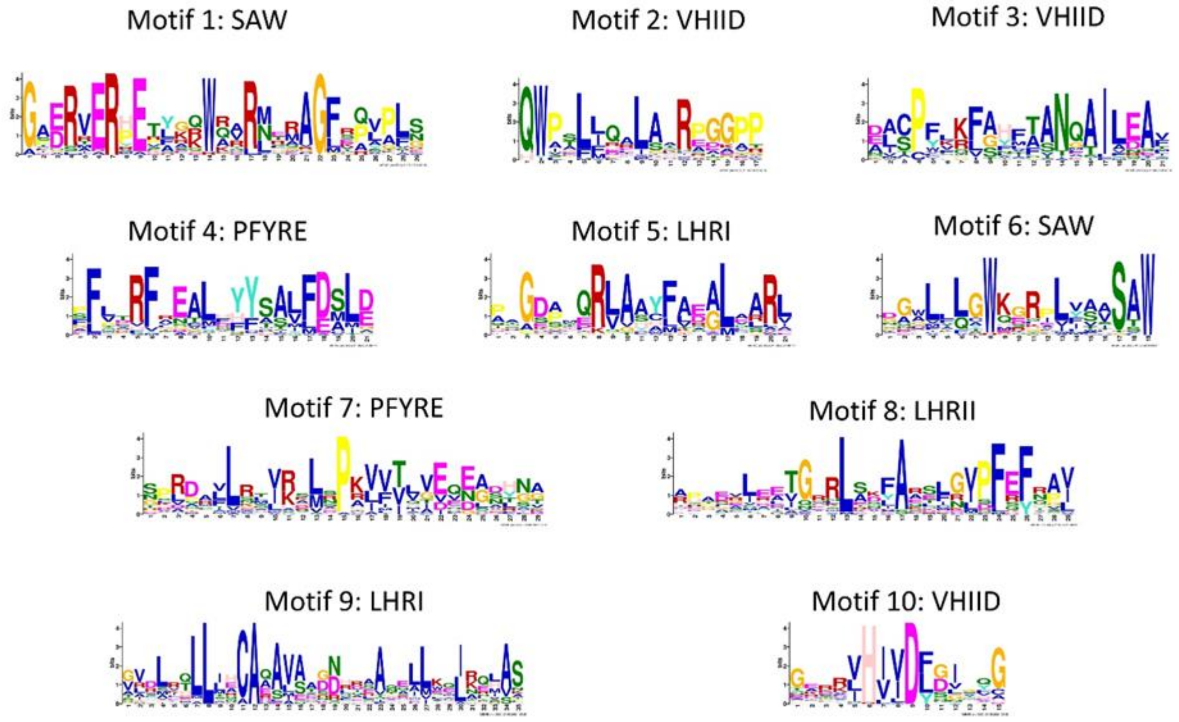

Supplementary Figure S2: Logos representing each MEME identified motif. A total of 10 motifs were identified, which were classified according to the conserved motifs of GRAS domain. Each logo represents the conserved sequence of the corresponding motif. The height of each amino acid in a logo indicates its frequency at that position.

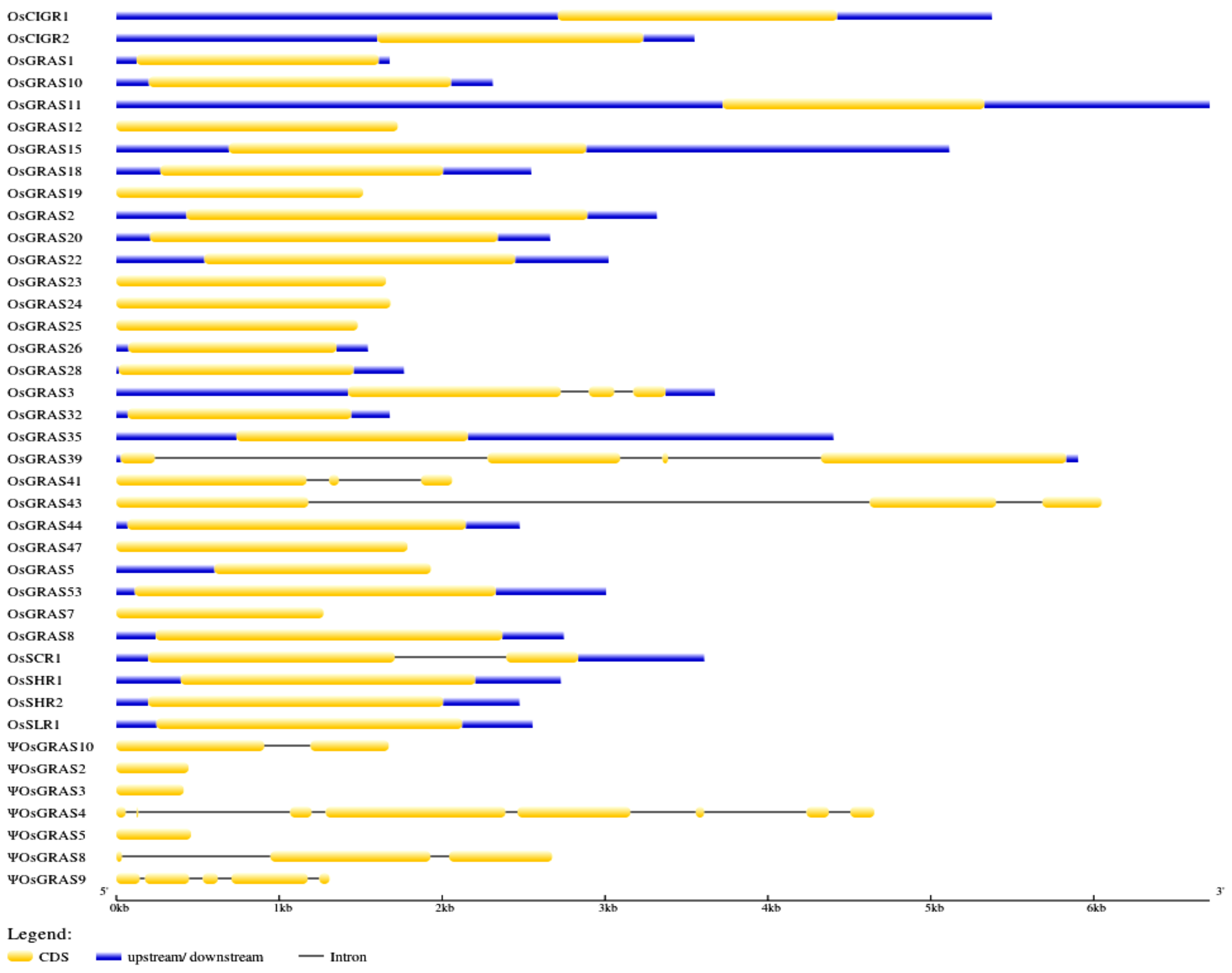

Supplementary Figure S3: Figure representing the genetic organisation of rice GRAS genes under study (developed through Gene Structure Display Server (GSDSv2)). The yellow coded region indicates the coding sequence and the blue region indicates the untranslated regions. The black line corresponds to the intronic sequences.

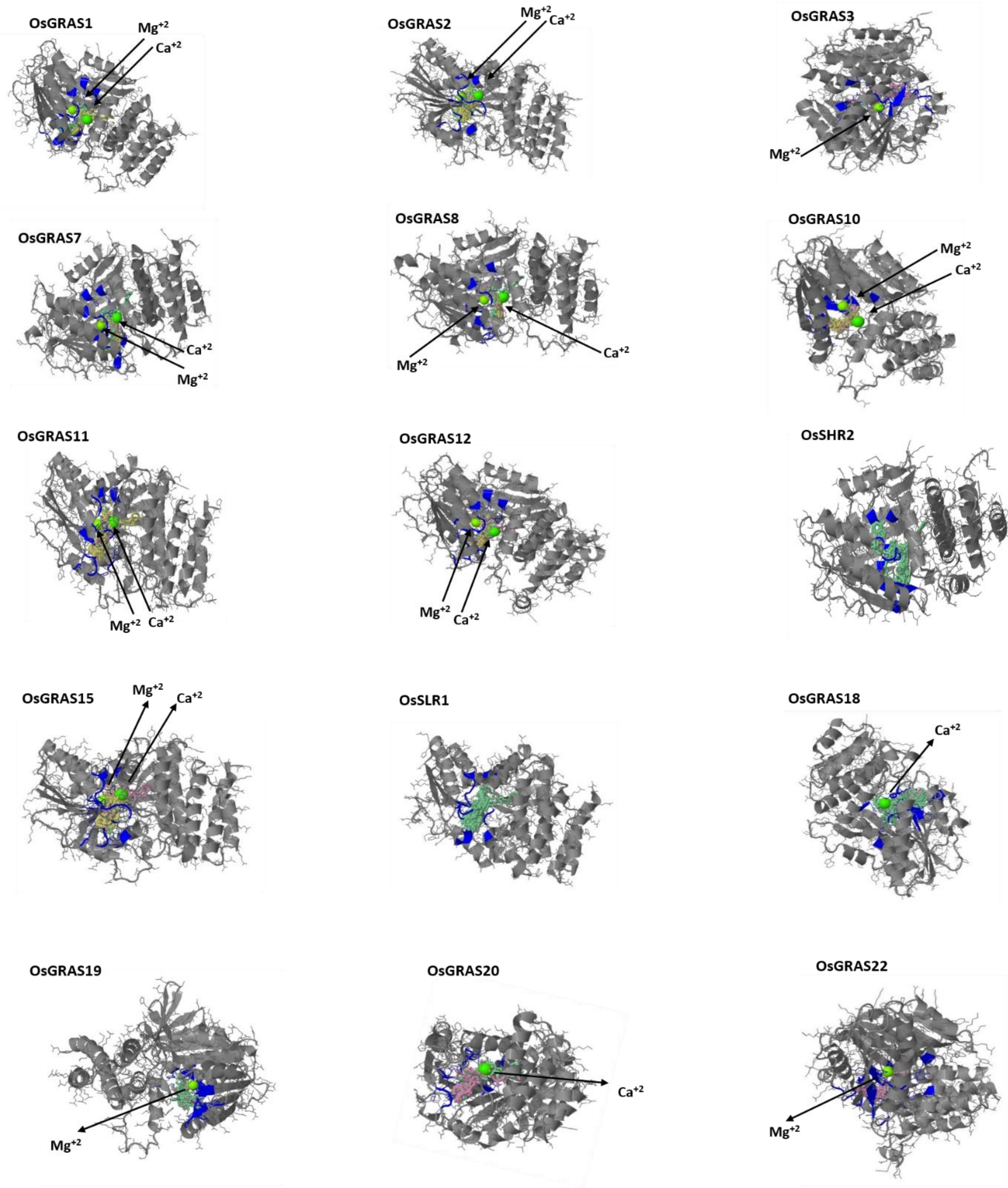
OsGRAS1
Mg+2
Ca+2
OsGRAS2
Mg+2
Ca+2
OsGRAS3
Mg+2
OsGRAS7
Ca+2
Mg+2
OsGRAS8
Mg+2
Ca+2
OsGRAS10
Mg+2
Ca+2
OsGRAS11
Mg+2
Ca+2
OsGRAS12
Mg+2
Ca+2
OsSHR2
OsGRAS15
Mg+2
Ca+2
OsSLR1
OsGRAS18
Ca+2
OsGRAS19
Mg+2
OsGRAS20
Ca+2
OsGRAS22
Mg+2

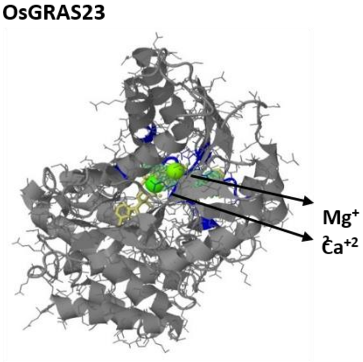

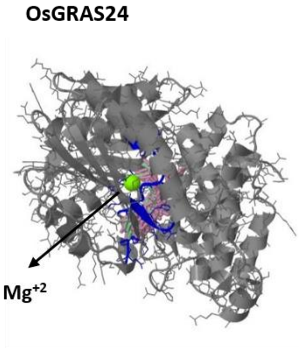

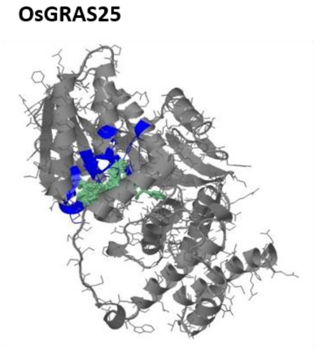

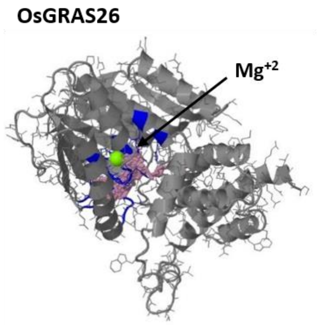

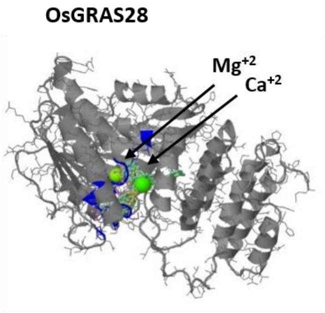

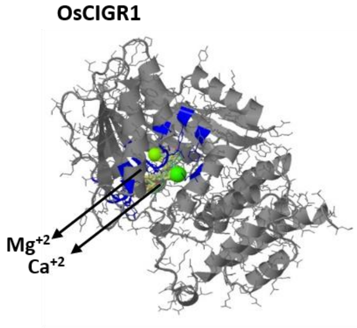

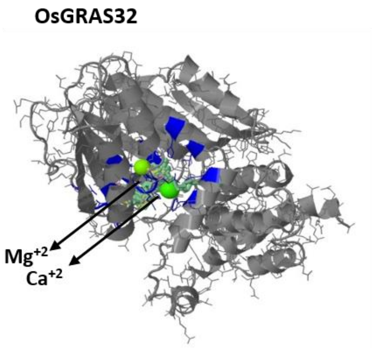

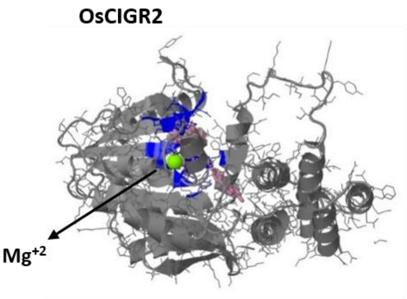

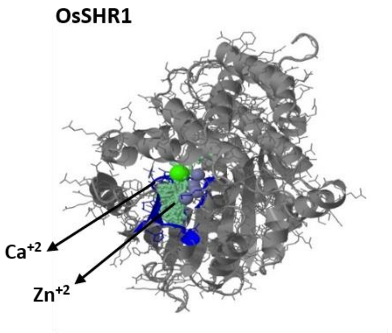

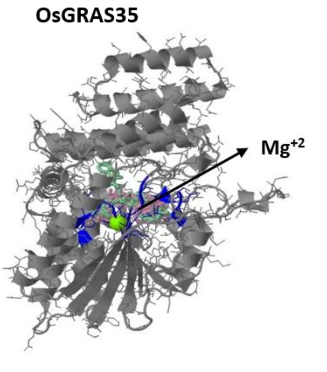

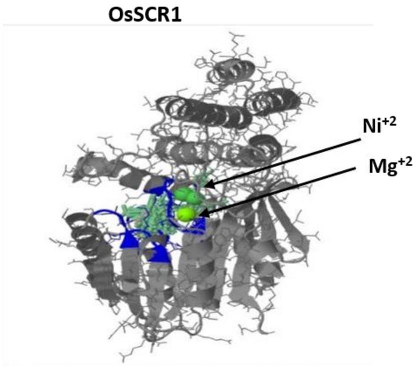

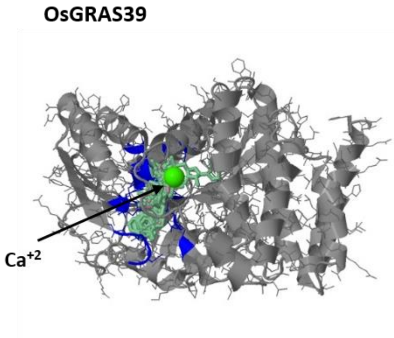

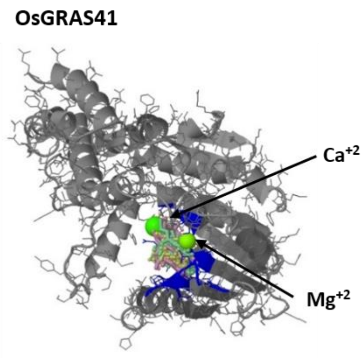

Supplementary Figure S4: Three dimensional structures of 28 GRAS proteins along with their interacting ligands as predicted by 3Dligand site and Phyre2 program. The blue labeled region indicates the point of interaction with metallic and non-metallic ligands. The metallic ligands are labeled. These include Ca^{+2}, Mg^{+2}, Ni^{+2}, Zn^{+2}.

I.A) 15min shoot (upregulated genes)

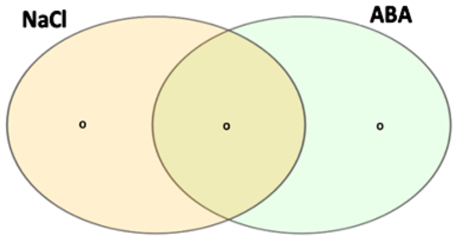

Treatment	Number of genes	Name of genes
NaCl	0	-
ABA	0	-
NaCl+ABA	0	-

I.B) 3h shoot (upregulated genes)

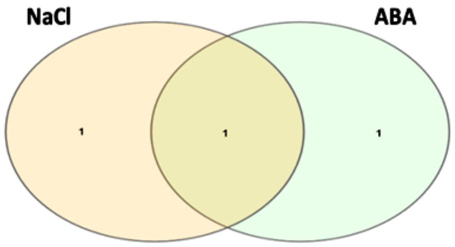

Treatment	Number of genes	Name of genes
NaCl	1	OsGRAS19
ABA	1	OsGRAS25
NaCl+ABA	1	ΨOsGRAS5

I.C) 12h shoot (upregulated genes)

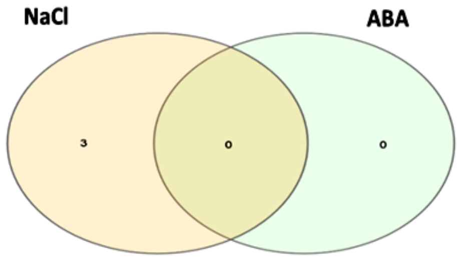

Treatment	Number of genes	Name of genes
NaCl	3	OsGRAS12,ΨOsGRAS5, OsGRAS25
ABA	0	-
NaCl+ABA	0	-

I.D) 24h shoot (upregulated genes)

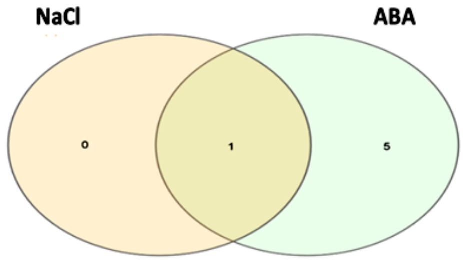

Treatment	Number of genes	Name of genes
NaCl	0	
ABA	5	ΨOsGRAS2,OsGRAS12 OsGRAS24,OsGRAS25 OsSCR1
NaCl+ABA	1	ΨOsGRAS5

I.E) 60h shoot (upregulated genes)

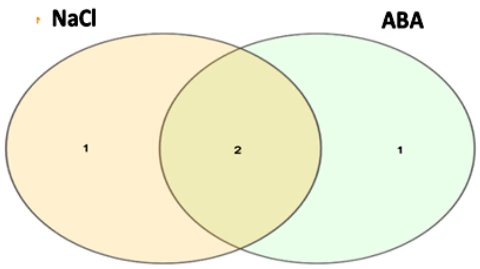

Treatment	Number of genes	Name of genes
NaCl	1	OsGRAS12
ABA	1	OsGRAS25
NaCl+ABA	2	ΨOsGRAS5 OsGRAS24

II.A) 15min root (upregulated genes)

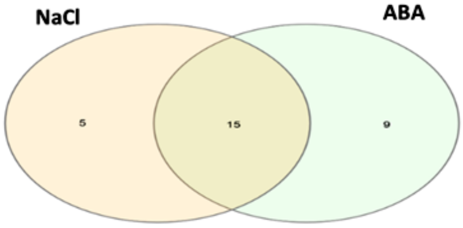

Treatment	Number of genes	Name of genes
NaCl	5	OsGRAS8,OsGRAS11,OsGRAS19,OsGRAS22,OsGRAS26
ABA	9	OsGRAS2,ΨOsGRAS2,ΨOsGRAS3,OsGRAS18,OsGRAS20,OsGRAS25,OsGRAS32,OsGRAS35,OsSCR1
NaCl+ABA	15	OsGRAS5,OsGRAS10,OsGRAS12,OsSHR2,ΨOsGRAS4,OsGRAS15,OsSLR1,ΨOsGRAS5,OsGRAS24,OsCIGR1,OsSHR1,OsGRAS39,OsGRAS41,OsGRAS43,OsGRAS44

II.B) 3h root (upregulated genes)

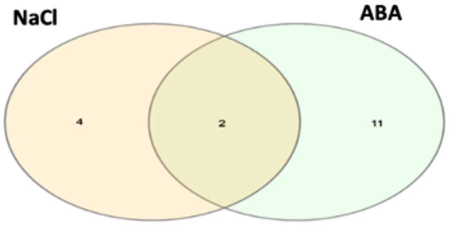

Treatment	Number of genes	Name of genes
NaCl	4	OsGRAS10,ΨOsGRAS3,ΨOsGRAS4,OsSHR1
ABA	11	OsGRAS5,ΨOsGRAS2,OsGRAS8,OsGRAS12,OsGRAS15,OsGRAS18,OsGRAS25,OsGRAS35,OsSCR1,OsGRAS41,OsGRAS44
NaCl+ABA	2	OsGRAS24,OsGRAS39

II.C) 12h root (upregulated genes)

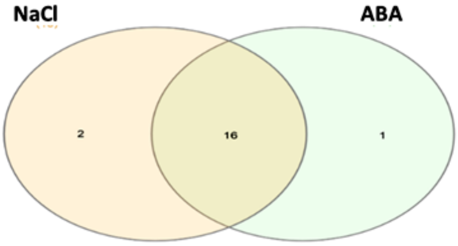

Treatment	Number of genes	Name of genes
NaCl	2	OsSHR2,OsGRAS19
ABA	1	OsGRAS41
NaCl+ABA	16	OsGRAS2,OsGRAS5,ΨOsGRAS2,OsGRAS8,OsGRAS10,OsGRAS12,OsGRAS15,OsSLR1,OsGRAS22,OsGRAS24,OsGRAS25,OsSHR1,OsGRAS35,OsSCR1,OsGRAS39,OsGRAS44

II.D) 24h root (upregulated genes)

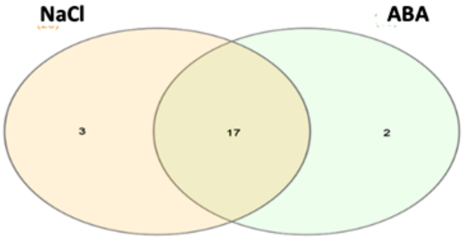

Treatment	Number of genes	Name of genes
NaCl	3	OsGRAS11,OsSHR2,ΨOsGRAS9
ABA	2	OsGRAS15,OsSHR1
NaCl+ABA	17	OsGRAS2,OsGRAS5,ΨOsGRAS2,OsGRAS8,OsGRAS10,OsGRAS12,OsSLR1,OsGRAS19,OsGRAS22,OsGRAS24,OsGRAS25,OsGRAS35,OsSCR1,OsGRAS39,OsGRAS41,OsGRAS43,OsGRAS44

II.E) 60h root (upregulated genes)

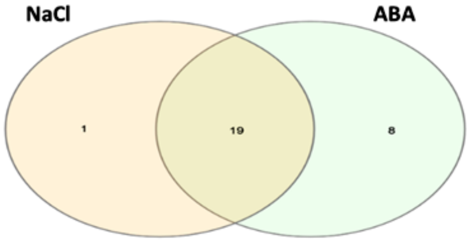

Treatment	Number of genes	Name of genes
NaCl	1	ΨOsGRAS3
ABA	8	OsGRAS2,OsGRAS3,OsGRAS18,OsGRAS20,OsGRAS22,OsGRAS41,ΨOsGRAS9,OsGRAS43
NaCl+ABA	19	OsGRAS5,ΨOsGRAS2,OsGRAS8,OsGRAS10,OsGRAS11,OsGRAS12,OsSHR2,OsGRAS15,OsSLR1,OsGRAS19,ΨOsGRAS5,OsGRAS24,OsGRAS25,OsGRAS26,OsSHR1,OsGRAS35,OsSCR1,OsGRAS39,OsGRAS44

III.A) 15min shoot (downregulated genes)

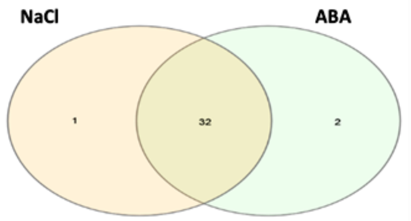

Treatment	Number of genes	Name of genes
NaCl	1	ΨOsGRAS2
ABA	2	ΨOsGRAS5,OsCIGR1
NaCl+ABA	32	OsGRAS8,OsGRAS10,OsGRAS11,OsGRAS12,OsSHR2, ΨOsGRAS3,ΨOsGRAS4,OsGRAS15,OsSLR1,OsGRAS18,O sGRAS19,OsGRAS20,OsGRAS22,OsGRAS23,OsGRAS24, OsGRAS25,OsGRAS26,OsGRAS28,OsGRAS32,OsCIGR2, OsSHR1,OsGRAS35,OsSCR1,ΨOsGRAS8,OsGRAS39, OsGRAS41,ΨOsGRAS9,OsGRAS43,OsGRAS44,OsGRAS47 ΨOsGRAS10,OsGRAS53

III.B) 3h shoot (downregulated genes)

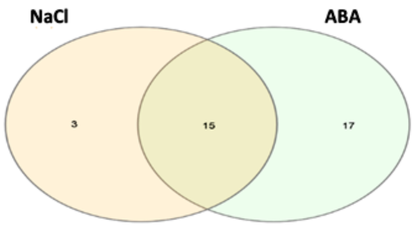

Treatment	Number of genes	Name of genes
NaCl	3	OsGRAS25,OsGRAS39,OsGRAS44
ABA	17	OsGRAS2,OsGRAS3,OsGRAS5,OsGRAS7,OsGRAS8, OsGRAS10,OsGRAS11,OsSHR2,ΨOsGRAS3,ΨOsGRAS4, OsGRAS15,OsSLR1,OsGRAS18,OsGRAS20,OsGRAS22, OsGRAS24,OsCIGR1
NaCl+ABA	15	OsGRAS23,OsGRAS26,OsGRAS28,OsGRAS32,OsCIGR2, OsSHR1,OsGRAS35,OsSCR1,ΨOsGRAS8,OsGRAS41, ΨOsGRAS9,OsGRAS43,OsGRAS47,ΨOsGRAS10,OsGRAS53

III.C) 12h shoot (downregulated genes)

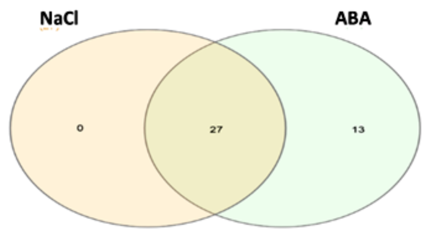

Treatment	Number of genes	Name of genes
NaCl	0	
ABA	13	OsGRAS1,OsGRAS2,OsGRAS3,OsGRAS5,OsGRAS12,OsG RAS19,ΨOsGRAS5,OsGRAS24,OsGRAS25, OsGRAS26,OsCIGR1,OsSCR1,ΨOsGRAS8
NaCl+ABA	27	OsGRAS7,ΨOsGRAS2,OsGRAS8,OsGRAS10,OsGRAS11, OsSHR2,ΨOsGRAS3,ΨOsGRAS4,OsGRAS15,OsSLR1, OsGRAS18,OsGRAS20,OsGRAS22,OsGRAS23,OsGRAS28 ,OsGRAS32,OsCIGR2,OsSHR1,OsGRAS35,OsGRAS39,Os GRAS41,ΨOsGRAS9,OsGRAS43,OsGRAS44,OsGRAS47, ΨOsGRAS10,OsGRAS53

III.D) 24h shoot (downregulated genes)

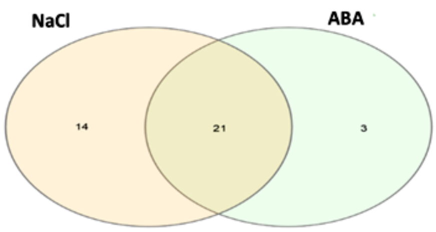

Treatment	Number of genes	Name of genes
NaCl	14	ΨOsGRAS2,OsGRAS8,OsGRAS10,OsGRAS11,Os GRAS12,OsSHR2,OsSLR1,OsGRAS19,OsGRAS24, OsGRAS25,OsGRAS26,OsSCR1,OsGRAS39,OsGR AS44
ABA	3	OsGRAS2,OsGRAS3,OsGRAS5
NaCl+ABA	21	OsGRAS7,ΨOsGRAS3,ΨOsGRAS4,OsGRAS15,Os GRAS18,OsGRAS20,OsGRAS22,OsGRAS23,OsGR AS28,OsCIGR1,OsGRAS32,OsCIGR2,OsSHR1, OsGRAS35,ΨOsGRAS8,OsGRAS41,ΨOsGRAS9, OsGRAS43,OsGRAS47,ΨOsGRAS10,OsGRAS53

III.E) 60h shoot (downregulated genes)

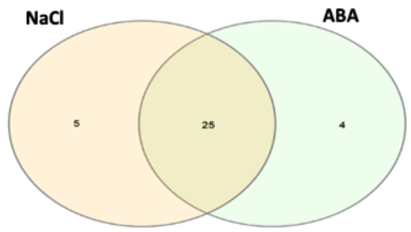

Treatment	Number of genes	Name of genes
NaCl	5	ΨOsGRAS2,ΨOsGRAS3,OsGRAS25,ΨOsGRAS8,OsGRAS44
ABA	4	OsGRAS2,OsGRAS3,OsGRAS10,OsSLR1
NaCl+ABA	25	OsGRAS7,OsGRAS8,OsGRAS11,OsSHR2,ΨOsGRAS4,OsGRAS15, OsGRAS18,OsGRAS19,OsGRAS20,OsGRAS22,OsGRAS23, OsGRAS26,OsGRAS28,OsCIGR1,OsGRAS32,OsCIGR2,OsSHR1, OsGRAS35,OsGRAS39,OsGRAS41,ΨOsGRAS9,OsGRAS43, OsGRAS47,ΨOsGRAS10,OsGRAS53

IV.A) 15min root (downregulated genes)

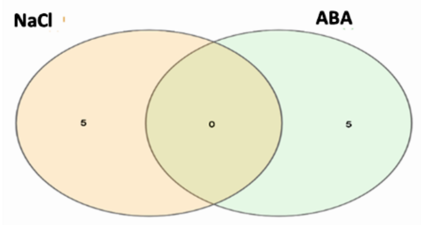

Treatment	Number of genes	Name of genes
NaCl	5	ΨOsGRAS3,OsGRAS25,OsGRAS28 OsSCR1,ΨOsGRAS8
ABA	5	OsGRAS1,OsGRAS3,OsGRAS7 OsGRAS8,OsGRAS19
NaCl+ABA	0	

IV.B) 3h root (downregulated genes)

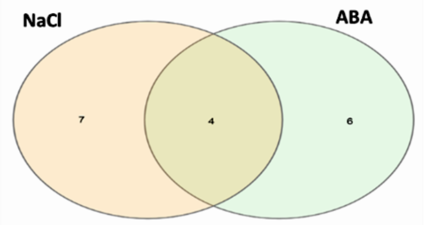

Treatment	Number of genes	Name of genes
NaCl	7	OsGRAS1,OsGRAS3,OsGRAS11,ΨOsGRAS5, OsGRAS28,OsCIGR1,OsGRAS32
ABA	6	OsGRAS7,OsSHR2,OsCIGR2,ΨOsGRAS8, OsGRAS43,ΨOsGRAS10
NaCl+ABA	4	OsGRAS2,OsGRAS20,OsGRAS23,OsGRAS26

IV.C) 12h root (downregulated genes)

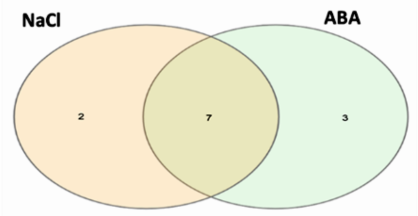

Treatment	Number of genes	Name of genes
NaCl	2	OsCIGR1,ΨOsGRAS9
ABA	3	ΨOsGRAS3,ΨOsGRAS4,OsGRAS28
NaCl+ABA	7	OsGRAS7,OsGRAS23,OsGRAS32,OsCIGR2,Ψ OsGRAS8,ΨOsGRAS10,OsGRAS53

IV.D) 24h root (downregulated genes)

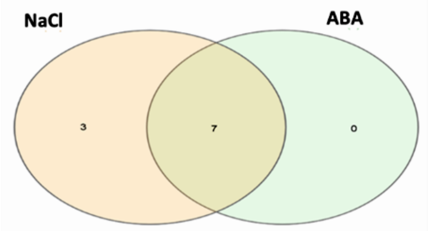

Treatment	Number of genes	Name of genes
NaCl	3	OsGRAS7,ΨOsGRAS3,ΨOsGRAS4
ABA	0	--
NaCl+ABA	7	OsGRAS23,OsGRAS28,OsGRAS32,OsCIGR2, ΨOsGRAS8,ΨOsGRAS10,OsGRAS53

IV.E) 60h root (downregulated genes)

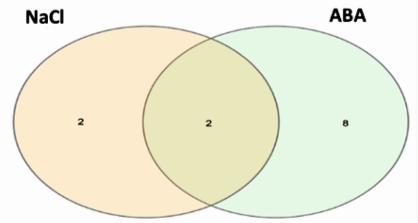

Treatment	Number of genes	Name of genes
NaCl	2	OsCIGR1,ΨOsGRAS9
ABA	8	ΨOsGRAS3,ΨOsGRAS4,OsGRAS23,OsGRAS28, OsGRAS32,OsCIGR2,ΨOsGRAS10,OsGRAS53
NaCl+ABA	2	OsGRAS7,ΨOsGRAS8

Supplementary Figure S5: Venn diagrams using interactiVenn software (Heberle et al., 2015) showing the number of genes upregulated in shoot (I.A-E) and root (II.A-E) or downregulated in shoot (III. A-E) and root (IV. A-E) during the course of treatment with NaCl and ABA. The corresponding number and the list of genes under each treatment and in combination is mentioned in the figure.

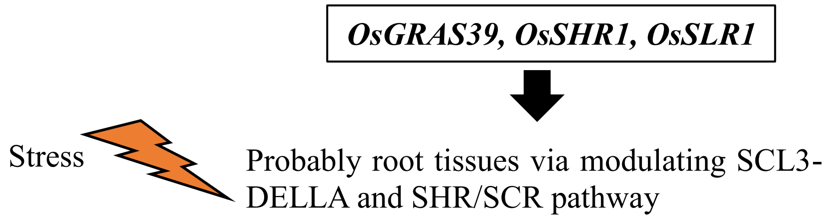

Supplementary Figure S6: Schematic diagram indicating the probable site of action of candidate genes during stress conditions.

Supplementary Table S1: List of genes and their organization details

Locus id	Gene name	Subfamily (according to Cenci and Rouard, 2017)	Chromosome number	Location	Gene size	Orientation	Splice forms	Introns	Exons
LOC_Os01g45860	OsGRAS1	OG-DELLA-2	Chr1	26045843 - 26044166	1488	3'-5'	1	0	1
LOC_Os01g62460	OsGRAS2	OG-LISCL	Chr1	36158308 - 36161536	2463	5'-3'	2	0	1
LOC_Os01g65900	OsGRAS3	OG-PAT-3	Chr1	38265509 - 38261836	1662	3'-5'	1	2	3
LOC_Os01g71970	OsGRAS5	OG-SCL3	Chr1	41711881 - 41713811	1329	5'-3'	1	0	1
LOC_Os02g10360	OsGRAS7	OG-LS	Chr2	5453090 - 5451819	1272	3'-5'	1	0	1
LOC_Os02g21685	ΨOsGRAS2	OG-PAT-4	Chr2	12888147 - 12887704	444	3'-5'	1	0	1
LOC_Os02g44360	OsGRAS8	OG-HAM-II	Chr2	26841585 - 26844331	2130	5'-3'	1	0	1
LOC_Os02g45760	OsGRAS10	OG-PAT-4	Chr2	27856163 - 27853853	1857	3'-5'	1	0	1
LOC_Os03g09280	OsGRAS11	OG-PAT-1	Chr3	4847121 - 4853832	1608	5'-3'	3	0	1
LOC_Os03g15680	OsGRAS12	OG-NSP2-1	Chr3	8651497 - 8653224	1728	5'-3'	1	0	1
LOC_Os03g31880	OsSHR2	OG-SHR-1	Chr3	18240730 - 18238256	1812	3'-5'	1	0	1
LOC_Os03g37900	ΨOsGRAS3	OG-DLT	Chr3	21050297 - 21050710	414	5'-3'	1	0	1
LOC_Os03g40080	ΨOsGRAS4	close to LISCL (unclassified)	Chr3	22262511 - 22267163	2334	5'-3'	1	7	8
LOC_Os03g48450	OsGRAS15	OG-LISCL	Chr3	27592832 - 27588776	2196	3'-5'	4	0	1
LOC_Os03g49990	OsSLR1	OG-DELLA-1	Chr3	28512625 - 28515179	1878	5'-3'	1	0	1
LOC_Os03g51330	OsGRAS18	OG-SCL4/7	Chr3	29370719 - 29373265	1737	5'-3'	1	0	1
LOC_Os04g35250	OsGRAS19	OG-SCLA	Chr4	21425721 - 21424207	1515	3'-5'	1	0	1
LOC_Os04g37440	ΨOsGRAS5	OG-DLT	Chr4	22312712 - 22312254	459	3'-5'	1	0	1
LOC_Os04g46860	OsGRAS20	OG-HAM-II	Chr4	27764666 - 27767328	2136	5'-3'	1	0	1
LOC_Os04g50060	OsGRAS22	OG-LISCL	Chr4	29857542 - 29860559	1911	5'-3'	2	0	1
LOC_Os05g31380	OsGRAS23	OG-SCL3	Chr5	18234846 - 18236501	1656	5'-3'	1	0	1
LOC_Os05g31420	OsGRAS24	OG-SCL3	Chr5	18275763 - 18277445	1683	5'-3'	1	0	1
LOC_Os05g40710	OsGRAS25	OG-SCR-3	Chr5	23871516 - 23872997	1482	5'-3'	1	0	1
LOC_Os05g42130	OsGRAS26	OG-SCL32-2	Chr5	24631172 - 24632715	1278	5'-3'	1	0	1
LOC_Os06g01620	OsGRAS28	OG-HAM-II	Chr6	365301 - 367066	1443	5'-3'	1	0	1
LOC_Os07g36170	OsCIGR1	OG-PAT-2	Chr7	21616338 - 21620316	1716	5'-3'	2	0	1
LOC_Os07g38030	OsGRAS32	OG-SCR-2	Chr7	22807706 - 22809384	1374	5'-3'	1	0	1
LOC_Os07g39470	OsCIGR2	OG-PAT-1	Chr7	23650525 - 23654073	1635	5'-3'	1	0	1
LOC_Os07g39820	OsSHR1	OG-SHR-1	Chr7	23868417 - 23871145	1809	5'-3'	1	0	1
LOC_Os07g40020	OsGRAS35	OG-SCL32-1	Chr7	24014021 - 24018422	1422	5'-3'	1	0	1
LOC_Os11g03110	OsSCR1	OG-SCR-1	Chr11	1119742 - 1123350	1956	5'-3'	1	1	2
LOC_Os11g04400	ΨOsGRAS8	OG-LISCL	Chr11	1829394 - 1832069	1650	5'-3'	1	2	3
LOC_Os11g04570	OsGRAS39	OG-SCL3	Chr11	1939818 - 1933914	2565	5'-3'	1	3	4
LOC_Os11g06180	OsGRAS41	OG-NSP2-2	Chr11	2950162 - 2952223	1419	5'-3'	1	2	3

LOC_Os11g11600	ΨOsGRAS9	close to LISCL (unclassified)	Chr11	6452428 - 6453736	1038	5'-3'	1	4	5
LOC_Os11g31100	OsGRAS43	OG-RAM1	Chr11	18102879 - 18096831	2319	3'-5'	1	2	3
LOC_Os11g47870	OsGRAS44	OG-LISCL	Chr11	28870096 - 28872571	2079	5'-3'	1	0	1
LOC_Os11g47910	OsGRAS47	OG-LISCL	Chr11	28895187 - 28896974	1788	5'-3'	1	0	1
LOC_Os12g06540	ΨOsGRAS10	OG-NSP2-2	Chr12	3170547 - 3172219	1389	5'-3'	1	1	2
LOC_Os12g38490	OsGRAS53	OG-LISCL	Chr12	23634672 - 23637678	2217	5'-3'	1	0	1

Supplementary Table S2: Chart depicting the protein properties of 40 GRAS genes under study. This includes their length, molecular weight, isoelectric points (*p*I), GRAVY indices, chelating ligands, low complexity region (LCR), localisation and secondary structure details

NAME	LENGTH (aa)	MW (KDa)	*p*I	GRAVY index	Ligand residues	Metallic & non-metallic ligands	LCR	TargetP-2.0 prediction	Disordered %	α- helix %	β-sheet %
OsGRAS1	495	52067.35	5.06	-0.017	LEU168 HIS172 ASP193 PHE194 SER195 LEU196 MET197 GLN198 GLN201 ILE224 GLY225 PRO226 PRO229 SER262 LEU263 ASP264 VAL266 VAL284 GLN286 ARG289 LEU290	Mg+2, SAM, Ca+2, SAH	Three (26-38,41-82,452-486)	-	30	40	11
OsGRAS2	820	90671.06	5.7	-0.415	LYS530 HIS534 ASP555 TYR556 GLY557 ILE558 TYR559 TYR560 GLN563 ILE586 ASP587 THR588 PRO589 GLN590 GLY592 SER624 ARG625 PHE626 GLU627 VAL629 MET647 LYS649 ASN652	Mg+2, SAM, Ca+2, SAH	Four (85-97, 165-183,246-262,415-434)	-	45	39	7
OsGRAS3	553	61798.49	4.8	-0.358	MET260 PHE270 PHE274 ASP295 PHE296 ASP297 ILE298 ASN299 GLN300 GLN303 VAL326 ASP327 ASP328 ALA364 ASN365 ILE366 GLY367 VAL369 ALA387 GLN389 HIS392	Mg^{+2}, SAM, NAP, SAH	Two (91-107,183-553)	-	35	39	10
OsGRAS5	442	48023.77	6.15	-0.079	LEU139 ASP164 LEU165 GLY166 GLY167 ALA168 ASP169 GLN172 VAL195 HIS196 GLU197 ARG228 LEU229 ASP230 SER250 GLN252	Mg^{+2}, SAM, Ca^{+2}, SAH	Four (46-73,89-105,260-270,367-383)	-	24	45	12
OsGRAS7	423	44138.74	5.56	-0.113	LEU122 HIS126 ASP153 LEU154 ASP155 ALA156 ALA157 HIS158 GLN161 ALA188 GLY189 THR190 LEU226 ALA227 VAL247 PHE249 LYS252	Mg+2, SAM, Ca+2, SAH	Two (21-29,39-420)	-	22	44	12
ΨOsGRAS2	147	15305.11	9.18	-0.128			Three (38-62,81-91,131-144)	-	48	58	0
OsGRAS8	709	74248.23	5.65	-0.02	LEU434 ASP461 PHE462 ASP463 GLY465 VAL466 GLN469 PHE496 MET497 LEU532 ASP533 ALA534 PHE535 PRO554	Mg+2, SAM, Ca+2, SAH	Five (47-85,88-106,153-165,195-217,258-273)	-	51	31	8
OsGRAS10	618	64200.52	9.27	-0.115	PHE335 ASP360 PHE361 ASP362 VAL363 SER364 GLN367 VAL390 ALA391 ASP392 CYS430 ARG431 ALA432 PRO433 ILE435 ALA453 THR455 ARG458	SAM, Mg+2, Ca+2,SAH	Six (23-35,40-50,62-72,98-116,143-160,162-179)	-	38	45	9
OsGRAS11	535	59647.91	5.86	-0.375	MET242 PHE252 TYR256 ASP277 PHE278 GLN279 ALA281 GLN282 ILE308 ASP309 ASP310 HIS315 ALA346 ALA347 SER348 HIS349 VAL351 ALA369 TYR370 GLN371 HIS374	Mg^{+2}, SAM, Ca^{+2}, SAH	One (80-93)	-	35	40	10
OsGRAS12	575	60848.2	5.04	-0.106	MET231 HIS235 ASP256 TYR257 ASP258 ILE259 ALA260 GLU261 GLN264 VAL289 SER290 ARG291 GLY294 GLY295 LEU325 ASP328 VAL349 LEU350 HIS351	Mg+2, SAM, Ca+2, SAH	Three (15-34,55-65,100-116)	-	35	43	9
OsSHR2	603	64247.77	5.93	-0.354	THR278 HIS282 LEU323 SER324 ASN325 THR326 PHE327 THR329 VAL354 VAL355 PRO356 THR357 HIS393 GLY395 ASP396 LEU397 VAL421 ASN422	SAM, SAH, NAD, ATP	Four (11-18,31-94,118-141,162-185)	-	41	36	9
ΨOsGRAS3	137	15192.7	7.87	-0.07			--	-	31	71	0
ΨOsGRAS4	777	88331.49	5.53	-0.486			--	-	40	34	9
OsGRAS15	731	82218.82	6.44	-0.483	LYS443 HIS447 ASP468 PHE469 GLY470 ILE471 TYR472 PHE473 ILE499 ASP500 VAL501 PRO502 GLN503 GLY505 PRO508 LYS538 TRP539 GLU540 ILE542 LEU560 ARG562 ASN565	Mg+2, SAM, Ca+2, SAH	--	-	40	39	8
OsSLR1	625	65406.24	5.14	-0.111	LEU323 HIS327 ASP348 PHE349 GLY350 ILE351 LYS352 GLN353 GLN356 VAL379 GLY380 PRO381 PRO382 GLN383 PRO384 ASP385 THR417 LEU418 ALA419 VAL446 PHE447 GLU448 ARG451	SAM, SAH	Four (9-17,128-140,185-207,209-232)	-	40	44	9

OsGRAS18	578	62477.53	5.63	-0.209	TYR283 HIS297 ASP318 PHE319 GLY320 ILE321 VAL322 VAL350 PRO351 SER352 PRO353 LEU354 LEU355 VAL390 MET411 LEU412 GLN413 TYR415 HIS416	SAM, Ca+2,SAH	One (144-206)	-	36	44	10
OsGRAS19	504	52579.87	8.67	-0.224	TYR169 TYR175 HIS179 ASP202 PHE203 ASP204 VAL205 SER206 TYR207 GLN210 PHE252 GLY253 ALA254 ASN285 ASN286 GLY287 SER288 THR290 VAL308	Mg+2, SAM, SAH	One (10-42)	-	31	45	11
ΨOsGRAS5	152	16672.18	9.43	-0.472			Two (104-124,130-146)	-	38	38	14
OsGRAS20	711	74007.88	5.57	0.004	LEU437 ASP464 PHE465 ASP466 LEU467 GLY468 VAL469 GLN472 PHE499 VAL500 SER501 LEU535 ASP536 ALA537 PRO557 VAL558	SAM, NAP, Ca+2, SAH	Eight (52-70,79-92,98-114,185-215,221-234,243-255,277-286,302-312)	-	52	32	8
OsGRAS22	636	71655.98	5.51	-0.424	PHE338 LYS348 ASP373 TYR374 GLY375 ILE376 GLN377 TYR378 VAL404 ASP405 LEU406 ARG412 ALA442 LYS443 TRP444 ASP445 ILE447 LEU467 ARG469 ASN472 MET473	Mg+2, SAM, SAH	Three (2-15,56-76,237-251)	-	37	38	8
OsGRAS23	551	56280.97	5.41	0.197	VAL182 ASP208 LEU209 GLY210 GLY211 GLY212 VAL213 ASP214 GLN217 VAL241 ASN242 GLU243 SER274 ILE275 GLU276 ASN303 GLN305 ARG308	Mg+2, SAM, Ca+2, SAH	Three (6-27,51-59,513-541)	-	36	41	10
OsGRAS24	560	58183.56	7.85	0.182	LEU248 ASP273 LEU274 GLY275 GLY276 ILE277 HIS278 HIS281 VAL305 HIS306 GLU307 SER338 VAL339 GLU340 THR360 GLN362 ARG365	Mg+2,SAM, SAH	Four (5-20,32-52,55-83,89-141)	-	42	36	9
OsGRAS25	493	51644.41	5.75	0.008	TYR207 ASP228 LEU229 ASP230 VAL231 VAL232 PRO233 GLY234 GLN238 PHE261 GLY262 MET263 ARG294 PRO295 GLY296 ALA298 TRP317 LEU318 ARG319 HIS320	SAM, SAH	Two (13-36,98-111)	-	30	45	10
OsGRAS26	425	45430.82	5.81	0.017	HIS131 PHE135 ASP156 LEU157 SER158 VAL159 THR160 HIS161 GLN164 PRO187 SER188 VAL189 ARG190 PRO191 ALA192 SER226 ALA227 THR228 THR229 GLN266 SER267 TRP268	SAM, Mg+2,SAH	One (9-20)	-	21	50	12
OsGRAS28	480	51017.01	5.46	-0.058	VAL213 ASP238 PHE239 ASP240 VAL241 GLY242 PHE243 LEU273 VAL274 SER275 PRO276 GLY277 SER278 SER279 PHE309 THR334	Mg+2, SAM, Ca+2, SAH	One (35-66)	-	36	45	11
OsCIGR1	571	64602.19	5.87	-0.478	MET278 PHE288 TYR292 ASP313 PHE314 GLN315 ILE316 ALA317 GLN318 GLN321 ILE344 ASP345 ASP346 VAL382 TYR383 ALA384 THR385 VAL387 THR405 GLN407	Mg+2, SAM, Ca+2, SAH	--	-	40	36	9
OsGRAS32	457	49096.59	5.51	-0.127	PHE148 ASN152 VAL158 HIS162 ASP183 LEU184 ASP185 ILE186 MET187 GLN188 GLN191 LEU214 GLY215 ALA216 LYS247 ILE248 GLY249 VAL251 VAL269 TRP271 HIS274	Mg+2, SAM, Ca+2, SAH	Three (8-45,55-62,437-449)	-	27	47	14
OsCIGR2	544	60108.16	6	-0.324	MET251 PHE261 TYR265 ASP286 PHE287 HIS288 ILE289 SER290 GLN291 GLN294 ILE317 ASP318 ASP319 ILE355 SER356 GLY357 SER358 VAL360 THR378 GLU380 HIS383 ILE384	SAM, Mg+2, SAH	--	-	35	40	10
OsSHR1	602	64709.33	5.61	-0.4	HIS286 LEU316 SER317 ASN318 THR319 PHE320 THR322 VAL347 VAL348 SER349 ALA350 HIS388 GLY390 ASP391 LEU392 VAL416 ASN417 MET527	SAM, Ca+2,SAH, Zn+2	Three (11-40,46-80,122-147)	-	39	40	7
OsGRAS35	473	50596.45	5.31	0.022	TYR174 LEU196 SER197 THR198 THR199 HIS200 ALA226 ASP227 VAL228 ALA229 TYR241 THR266 SER274 LEU275 VAL276 HIS297 MET298 LEU299 THR302	Mg+2, SAM, SAH	Two (9-25,34-54)	-	27	43	11

OsSCR1	651	69918.23	5.91	-0.221	PHE364 HIS378 ASP399 LEU400 ASP401 ILE402 MET403 GLN407 LEU430 GLY431 ALA432 ASP462 LYS463 ALA464 LEU485 HIS487 LEU489 TYR490 ASP491 VAL492 THR493	$Ni^{+2,}$ Mg^{+2}, SAM, NAP, SAH	Six (3-55,86-101,115-134,148-157,188-229,235-279)	-	48	37	9
ΨOsGRAS8	549	59950.36	4.98	-0.299			Four (29-40,108-122,140-147,177-193)	-	34	48	8
OsGRAS39	854	94267.73	7.45	-0.09	LEU495 TYR499 ASP520 PHE521 SER522 GLY523 PRO524 ALA525 ALA526 ASN527 GLN530 VAL553 HIS554 ASP555 ALA585 LYS586 LEU587 ASP588 VAL612 GLN614 ARG617	SAM, SAH, Ca^{+2}	One (370-391)	Chloroplast	23	45	12
OsGRAS41	472	52312.55	4.78	0.111	VAL135 HIS139 ASP160 LEU161 ASN162 ILE163 GLY164 GLU165 GLN168 ILE189 THR190 THR191 VAL225 HIS226 ASN227 GLU228 GLU230 THR248 THR249 SER250	Mg+2, SAM, Ca+2, SAH	--	-	23	53	7
ΨOsGRAS9	345	37286.47	10.13	-0.552			One (216-254)	-	35	49	8
OsGRAS43	772	81094.36	6.18	-0.156			Eight (2-13,165-177,183-201,223-230,280-288,293-306,326-341,355-364)	-	39	36	11
OsGRAS44	692	77442.34	5.01	-0.394			Four (14-27,82-111,209-218,279-299)	-	39	43	8
OsGRAS47	595	66666.1	5.99	-0.372			One (201-211)	-	33	48	10
ΨOsGRAS10	462	50587.65	4.53	-0.137			Two (22-30,47-60)	-	44	44	7
OsGRAS53	738	81365.04	5.17	-0.305			Nine (53-65,116-135,150-162,241-260,337-356,796-808,859-878,893-905,984-1003)	-	39	40	7

Supplementary Table S3: List of domains observed in GRAS genes. The number, position and sequences of the observed domains and LCR are provided in the chart.

Name	Domain	Position	Sequence	LCR	Position	Sequence
OsGRAS1	GRAS	86-449	LVHLLMSCAGAIEAGDHALASAQLADSHAALAAVSAASGIGRVAVHFTTALSRRLFPSPVAPPTTDAEHAFLYHHFYEACPYLKFAHFTANQAILEAFHGCDHVHVIDFSLMQGLQWPALIQALALRPGGPPFLRITGIGPPSPTGRDELRDVGLRLADLARSVRVRFSFRGVAANSLDEVRPWMLQIAPGEAVAFNSVLQLHRLLGDPADQAPIDAVLDCVASVRPKIFTVIEQEADHNKTGFLDRFTEALFYYSAVFDSLDAASASGGAGNAMAEAYLQREICDIVCGEGAARRERHEPLSRWRDRLTRAGLSAVPLGSNALRQARMLVGLFSGEGHSVEEADGCLTLGWHGRPLFSASAWE	LCR1	26-38	PPPAAVAPDDGVG
				LCR2	41-82	DPPAGADVDAAALPEFAAAFPPCAPDAAAAVLAMRREEEEVA
				LCR3	452-486	GDGGGDNNNNSNSNVSGSSGSDSNNSGSSNGKSSG
OsGRAS2	GRAS	443-813	LETLLIHCAQSVATDDRRSATELLKQIRQHAHANGDGDQRLAHCFANGLEARLAGTGSQIYKNYTITRLPCTDVLKAYQLYLAACPFKKISHYFANQTILNAVEKAKKVHIVDYGIYYGFQWPCLIQRLSNRPGGPPKLRITGIDTPQPGFRPAERTEETGRYLSDYAQTFNVPFEFQAIASRFEAVRMEDLHIEEDEVLIVNCMFKFKNLMDESVVAESPRNMALKTIRKMNPHVFIHGVVNGSYNAPFFVTRFREALFHYSAIFDMLETNIPKDNEQRLLIESALFSREAINVISCEGLERMERPETYKQWQVRNQRVGFKQLPLNQDMMKRAREKVRCYHKDFIIDEDNRWLLQGWKGRILFALSTWK	LCR1	85-97	VSVSASAASSAAA
				LCR2	165-183	SSAANSCNSLSPCNCSSSS
				LCR3	246-262	SQSSSFASSNGSSVTFS
				LCR4	415-434	KHSGGGHGKGSSHGKGRGKK
OsGRAS3	GRAS	183-553	PKQLLFDCAMALSDYNVDEAQAIITDLRQMVSIQGDPSQRIAAYLVEGLAARIVASGKGIYKALSCKEPPTLYQLSAMQILFEICPCFRFGFMAANYAILEACKGEDRVHIIDFDINQGSQYITLIQFLKNNANKPRHLRITGVDDPETVQRTVGGLKVIGQRLEKLAEDCGISFEFRAVGANIGDVTPAMLDCCPGEALVVNFAFQLHHLPDESVSIMNERDQLLRMVKGLQPKLVTLVEQDANTNTAPFQTRFREVYDYYAALFDSLDATLPRESPDRMNVERQCLAREIVNILACEGPDRVERYEVAGKWRARMTMAGFTPCPFSSNVISGIRSLLKSYCDRYKFEEDHGGLHFGWGEKTLIVSSAWQ	LCR	91-107	SNVSQQNSQSISDNQSS
OsGRAS5	GRAS	50-437	LIHLLLNCAAAAAAGRLDAANAALEHIASLAAPDGDAMQRVAAAFAEALARRALRAWPGLCRALLLPRASPTPAEVAAARRHFLDLCPFLRLAGAAANQSILEAMESEKIVHVIDLGGADATQWLELLHLLAARPEGPPHLRLTSVHEHKELLTQTAMALTKEAERLDVPFQFNPVVSRLDALDVESLRVKTGEALAICSSLQLHCLLASDDDAAAVAGGDKERRSPESGLSPSTSRADAFLGALWGLSPKVMVVAEQEASHNAAGLTERFVEALNYYAALFDCLEVGAARGSVERARVERWLLGEEIKNIVACDGGERRERHERLERWARRLEGAGFGRVPLSYYALLQARRVAQGLGCDGFKVREEKGNFFLCWQDRALFSVSAWR			
OsGRAS7	GRAS	39-420	ARGLVLACADLVHRGDLDGARRVAEAVLAAADPRGEAGDRLAHHFARALLALRGGGKGGHGGGGGGVVPSSAAYLAYIKIAPFLRFAHLTANQAILEAAAADAGGAHRRVLHIVDLDAAHGVQWPPLLQAIADRADPAVGPPPEVRLTGAGTDRDVLLRTGDRLRAFSSSLNLPFRFHPLILPCTAELAADPTAALELHPDETLAVNCVLFLHKLGGDGELAAFLRWVKSMNPAVVTIAEREGVLGGDVDDDNVPDELPRRVAAAMDYYSSVFDALEATVPPASADRLAVEQEILSREIDAAVAAPGAGGGGRARDFDAWASAARAAGLAPRPLSAFAASQARLLLRLHYPSEGYKADDDGGRGACFLRWQQRPLMSVSSWQ	LCR	21-29	QPQPQPQPP
ΨOsGRAS2	GRAS	62-138	SRQLLSEAAAAIANGNHIVAASLLSALKLSVNPQGDAEQRLVAMMVAALSSCVGTSPSQHLADLYIGVGRRRWSEDR			
OsGRAS8	GRAS	349-708	LLDELAAAAKATEAGNSVGAREILARLNQQLPQLGKPFLRSASYLKEALLLALADSHHGSSGVTSPLDVALKLAAYKSFSDLSPVLQFTNFTATQALLDEIGGMATSCIHVIDFDLGVGGQWASFLQELAHRRGAGGMALPLLKLTAFMSTASHHPLELHLTQDNLSQFAAELRIPFEFNAVSLDAFNPAELISSSGDEVVAVSLPVGCSARAPPLPAILRLVKQLCPKVVVAIDHGGDRADLPFSQHFLNCFQSCVFLLDSLDAAGIDADSACKIERFLIQPRVEDAVIGRHKAQKAIAWRSVFAATGFKPVQLSNLAEAQADCLLKRVQVRGFHVEKRGAALTLYWQRGELVSISSWR	LCR1	47-85	SPSPPYSTSTLSSSLGGGSADSTGVAAVSESSTAAAGAT
				LCR2	88-106	GAPGEHGGGGKEEWGGGCE
				LCR3	153-165	QPGPPLPVLQQPL
				LCR4	195-217	SSSGAHTATGGGGKASLGFGLFS
				LCR5	258-273	PPNPAAALFMPLPPFP
OsGRAS10	GRAS	253-618	SRQLLSEAAAAVADGNHTAAASLLSALKLSANPRGDAEQRLVAMMVAALSSRVGTGPSQHLADLYSGEHRAACQLLQDVSPCFGLALHGANLAILDAVAGHRAIHLVDFDVSAAQHVALIKALADRRVPATSLKVTVVADPTSPFTPAMTQSLAATCERLKKLAQQAGIDFRFRAVSCRAPEIEASKLGCEPGEALAVNLAFTLSRVPDESVSPANPRDELLRRVRALGPRVVTLVEQELNTNTAPMAARFSDASAHYGAVLESLDATLGRDSADRTRAEAALASKVANAVGREGPDRVERCEVFGKWRARFGMAGFRAVAIGEDIGGRVRARLGPALPAFDVKLDNGRLGVGWMGRVVTVASAWR	LCR1	23-35	AALQAAARQQSQQ
				LCR2	40-50	GAGGAGVTGGV
				LCR3	62-72	QQQRQVAAQQA
				LCR4	98-116	GISGLSSGFGGISQQQPSS
				LCR5	143-160	TAQNQAVARAPAARPATA
				LCR6	162-179	ELVLLQELEKQLLGDDEE
OsGRAS11	GRAS	166-535	LKQVIAACGKAVDENSWYRDLLISELRNMVSISGEPMQRLGAYMLEGLVARLSSTGHALYKSLKCKEPTSFELMSYMHLLYEICPFFKFGYMSANGAIAEAVKGENFVHIIDFQIAQGSQWATMIQALAARPGGPPYLRITGIDDSNSAHARGGGLDIVGRRLFNIAQSCGLPFEFNAVPAASHEVMLEHLDIRSGEVIVVNFAYQLHHTPDESVGIENHRDRILRMVKGLSPRVVTLVEQEANTNTAPFFNRYLETLDYYTAMFEAIDVACPRDDKKRISTEQHCVARDIVNLIACEGAERVERHEPFGKWRARLSMAGFRPYPLSALVNNTIKKLLDSYHSYYKLEERDGALYLGWKNRKLVVSSAWR	LCR	80-93	HSSTSSHISGSPIS
OsGRAS12	GRAS	132-526	LLHLLMAAAEALSGPHKSRELARVILVRLKEMVSHTASANAAASNMERLAAHFTDALQGLLDGSHPVGGSGRQAAAAASHHHAGDVLTAFQMLQDMSPYMKFGHFTANQAILEAVSGDRRVHIVDYDIAEGIQWASLMQAMTSRADGVPAPHLRITAVSRSGGGGARAVQEAGRRLSAFAASIGQPFSFGQCRLDSDERFRPATVRMVKGEALVANCVLHQAAATTTIRRPTGSVASFLSGMAALGAKLVTVVEEEGEAEKDDDGDSAGDAAAGGFVRQFMEELHRYSAVWDSLEAGFPTQSRVRGLVERVILAPNIAGAVSRAYRGVDGEGRCGWGQWMRGSGFTAVPLSCFNHSQARLLLGLFNDGYTVEETGPNKIVLGWKARRLMSASVWA	LCR1	15-34	SGCGSTTTTSSASSLDDGTG
				LCR2	55-65	DDDGGHDLHGL
				LCR3	100-116	GNGSNPSSTTTTNPGSP
OsSHR2	GRAS	188-502	AAQLLMECARAVAGRDSQRVQQLMWMLNELASPYGDVDQKLASYFLQGLFARLTTSGPRTLRTLATASDRNASFDSTRRTALKFQELSPWTPFGHVAANGAILESFLEAAAAGAAASSSSSSSSTPPTRLHILDLSNTFCTQWPTLLEALATRSSDDTPHLSITTVVPTAAPSAAAQRVMREIGQRLEKFARLMGVPFSFRAVHHSGDLADLDLAALDLREGGATAALAVNCVNALRGVARGRDAFVASLRRLEPRVVTVVEEEADLAAPEADASSEADTDAAFVKVFGEGLRFFSAYMDSLEESFPKTSNERLSLERAVGRAIVDLVSCPASQSAERRETAASWARRMRSAGFSPAAFSEDVADDVRSLLRRYKEGWSMRDAGGATDDAAGAAAAGAFLAWKEQPVVWASAWK	LCR1	11-18	HHHHHHQH
				LCR2	31-94	SYPSSRGSTSSPSSHHTHNHTYYHHSHSHYNNNSNTNYYYQGGGGGGGGYYYAEEQQPAAYLEE
				LCR3	118-141	SGTGAPSSAPVPPPPSATTSSAGG
				LCR4	162-185	GGSPAVPSSSGAGAGAGAAPSSSG
ΨOsGRAS3	GRAS	38-137	LVRMLTACADSVSAGNHEAAIYYLARLCEMASLAGPMPIHRVAAYFIEVLTLRVVRMWPHMFNISPPRELTNDAFSGDDDAMALRILNTITPILLLGKHS			
ΨOsGRAS4	GRAS	360-435	LRTLLINCAQAVSVSNHSLASDILKIIRHHASPTGDDSQRLALCLAYCLDVRLTGTGSQIYHKFITKRRNVKDILK			
	GRAS	428-672	RNVKDILKVHIIDFGICFGFQWPSLFEELAKIEDGPPKLRITGIELPESGFRPYARSNNIGLRLADYAKTFNIPFEYQHISSNKWEALSPEDFNIEKDEVLIVNCIYRIKDLGDETISINSARSRVLNTIRMMKPKVFVQGVLNGSYGVPFFLTRFKEVMYHYNSLFDMLDKNIPRDNETRMIIERDIYQYIMLNVIACEGPERIERPESYKKWKVRNLKAGLVQLPLNPAIVRETQDMSSDKAS			
	RPT1	730-756	VSDDFYHVSGDTREISCDTYQVLDDFY			
	RPT1	751-777	VLDDFYHVSGDTYEISDDTYRISGDSY			
OsGRAS15	GRAS	356-727	LRTLLIHCAQAVAADDRRTANELLKQIRQHAKPNGDGSQRLAYCFADGLEARLAGTGSQLYHKLVAKRTTASDMLKAYHLYLAACPFKRLSHFLSNQTILSLTKNASKVHIIDFGIYFGFQWPCLIRRLFKREGGPPKLRITGIDVPQPGFRPTERIEETGQRLAEYAEKIGVPFEYQGIASKWETICVEDLNIKKDEVVIVNCLYRFRNLIDETVAIDSPRNRVLNTIRQVNPAIFIHGIVNGSYSVPFFITRFREALFHFSALFDMLETTVPRDDAQRALIERDLFGREALNVIACEGSDRVERPETYKQWQVRNLRAGFVQSPLNQDIVLKAKDKVKDIYHKDFVIDEDSEWLLQGWKGRIIYAISTWK	LCR1	50-64	SSAASSTASRAAVSS
				LCR2	133-148	PLDSPSESSTSSYPHS
OsSLR1	DELLA	39-120	DELLAALGYKVRSSDMADVAQKLEQLEMAMGMGGVSAPGAADDGFVSHLATDTVHYNPSDLSSWVESMLSELNAPLPPIPPA	LCR1	9-17	GGSSGGGSS
	GRAS	241-621	LVHALLACAEAVQQENFAAAEALVKQIPTLAASQGGAMRKVAAYFGEALARRVYRFRPADSTLLDAAFADLLHAHFYESCPYLKFAHFTANQAILEAFAGCHRVHVVDFGIKQGMQWPALLQALALRPGGPPSFRLTGVGPPQPDETDALQQVGWKLAQFAHTIRVDFQYRGLVAATLADLEPFMLQPEGEADANEEPEVIAVNSVFELHRLLAQPGALEKVLGTVHAVRPRIVTVVEQEANHNSGSFLDRFTESLHYYSTMFDSLEGGSSGQAELSPPAAGGGGGTDQVMSEVYLGRQICNVVACEGAERTERHETLGQWRNRLGRAGFEPVHLGSNAYKQASTLLALFAGGDGYRVEEKEGCLTLGWHTRPLIATSAWR	LCR2	128-140	STSSTVTGGGGSG
				LCR3	185-207	GGGSTSSSSSSSSSLGGGASRGS
				LCR4	209-232	VEAAPPATQGAAAANAPAVPVVVV
OsGRAS18	SCOP	15-95	QQVIQQQQQQQQQQQRHHHHHHLPPPPPPQSMAPHHHQQKHHHHHQQMPAMPQAPPSSHGQIPGQLAYGGGAAWPAGEHF	LCR1	144-206	TTPPPPVPSPPPTHAAATATATAATAAPRPEAAPALLPQPAAATPVACSSPSPSSADASCSAP
	GRAS	207-578	ILQSLLSCSRAAATDPGLAAAELASVRAAATDAGDPSERLAFYFADALSRRLACGTGAPPSAEPDARFASDELTLCYKTLNDACPYSKFAHLTANQAILEATGAATKIHIVDFGIVQGIQWAALLQALATRPEGKPTRIRITGVPSPLLGPQPAASLAAT			

			NTRLRDFAKLLGVDFEFVPLLRPVHELNKSDFLVEPDEAVAVNFMLQLYHLLGDSDELVRRVLRLAKSLSPAVVTLGEYEVSLNRAGFVDRFANALSYYRSLFESLDVAMTRDSPERVRVERWMFGERIQRAVGPEEGADRTERMAGSSEWQTLMEWCGFEPVPLSNYARSQADLLLWNYDSKYKYSLVELPPAFLSLAWEKRPLLTVSAWR			
OsGRAS19	GRAS	86-495	LVRLLLSAVAAGEAGDARAAAAALREVDRRASCRGGGDPAQRVAACYAAALAPRLAAGLRPARSSPAAPAAARAEQFLAYTMFYQASPFYQFAHFTANQAIVEAFESGGRRRLHVVDFDVSYGFQWPSLIQSLSDAAAAATSSSSHDDDDNGGGCGDGPVSLRITGFGASADELRETEARLRRFAAGCPNLRFEFEGILNNGSNTRHDCTRIDDDATVVVNLVFPASSREACAATRMAYINSLNPSMVFLIEKHDGGGGLTGGDNTTTGRSASLLPRFAANLRYFAAVFDSLHECLPADSAERLAIERDHLGREIADAVASLDHQHRRRHGGGGGGGDHAAASWNWKAAMEGAGLDGVKLSSRTVSQAKLLLKMKSGCGGGGFRVVEGDGGMAMSLAWRDMALATATLWR	LCR	10-42	DGGGGGDAAAAVAKKSKVVGGGAVVVDGVGSSA
ΨOsGRAS5	GRAS	1-80	MHYLRYYDAAFDAVDAAGLLETRPARAKVEEMFAREIRNAVAFEGAERFERHESFAGRRRRMEDGGGLQWGSKAEEKCLL	LCR1	104-124	SLPPAVAAAPLVLPLPRASAA
				LCR2	130-146	APMPPTAAPLVLPPPLP
OsGRAS20	GRAS	352-710	LLDELAAAAKATEVGNSIGAREILARLNQQLPPIGKPFLRSASYLKDALLLALADGHHAATRLTSPLDVALKLTAYKSFSDLSPVLQFANFTVTQALLDEIASTTASCIRVIDFDLGVGGQWASFLQELAHRCGSGGVSLPMLKLTAFVSAASHHPLELHLTQDNLSQFAADLGIPFEFNAINLDAFDPMELIAPTADEVVAVSLPVGCSARTPLPAMLQLVKQLAPKIVVAIDYGSDRSDLPFSQHFLNCLQSCLCLLESLDAAGTDADAVSKIERFLIQPRVEDAVLGRRRADKAIAWRTVLTSAGFAPQPLSNLAEAQADCLLKRVQVRGFHVEKRGAGLALYWQRGELVSVSAWR	LCR1	52-70	GSPSPPNSTSTLSSSHGSG
				LCR2	79-92	VAAVSESSAAAAEA
				LCR3	98-114	PGEHGGGGGGELPPIPG
				LCR4	185-215	SSPAALASDLSSSGGRSLTSSSGSNSKATSA
				LCR5	221-234	PEAALQPPPATTAP
				LCR6	243-255	PPLLGLPSPTLLL
				LCR7	277-286	QQQPLLQPPP
				LCR8	302-312	QPQPPPPAPAQ
OsGRAS22	GRAS	261-634	LTTLLIHCAQAAAIDDHRNSNELLKQIRQRSSAYGDAGQRLAHCFANALEARLAGTGSNIYRSLAAKRTSVYDILNAFKLYVTACPFKKISNFFSIEAILNASKGMTRLHIVDYGIQYGFQWPIFFQRISKRPGGPPSVRITGVDLPQPGFRPAQLIEATGRRLHDYARMFNVPFEYHAIAAKWDTIRVEDLKIDKDKDELLVVNCLFRMRNMMDEMVTDDSPRMQVLKTIRKMNPNLFIHGVVNGTYNAPFFVTRFKEALFYYSSLFDMLETTASRVDENRLLIERDLFGREALNVVACEGTERVERPETYKQWQVRNIRAGFKQLPLNQETVKKARYKVKKSYHRDFLVDEDNKWMLQGWKGRIIFALSAWE	LCR1	2-15	LDSGSYDDVDYGDL
				LCR2	56-76	STPSPTSTTTELENSEDLSES
				LCR3	237-251	KGSGNKRGRKKGKSG
OsGRAS23	GRAS	92-506	IAAFLADGTCQMQVNDGLSCVVDLAGGDADGGGVGEGRSAQRLASAFAEALALRFILPCDGVCRSLHLTRAPPPPAVSAARQGFRAMCPFVRLAAAAANLSIAEVMEAERAVVHVVDLGGGVDANQWVELVRLVAARPGGPPGLLRLTVVNESEDFLSAVAAYVAAEAQRLDLSLQFHPVLSSIEELSATATGSIGSRLVVIPGQPLAVVANLQIHRLLAFPDYVDGVASRRPAAEQSGSSQHTMTTATKTKADALLRAIRDLNPKLVVLTENEADHNVAELGARVWNALNYYAALFDALEASSTPPAAVPPHERACVERWVLGEEIKDIVVREGTGRRERHETLGRWAERMVAAGFSPVTAARALASTETLAQQMVAAGGGGAGAGVLRAAHGGGCFPVICWCDVPVFSVSTWT	LCR1	6-27	PRLALGGGGGGAGGERLPAAGE
				LCR2	51-59	AAMAAAAAA
				LCR3	513-541	PAPPLWPPAAAGGAGPSGSGYGGDGPSTA
OsGRAS24	GRAS	154-558	LHGHLRRCAEALAASRPADADAELASIARMASSDGDAVQRVAAAFAEAMARVVIRPWRGVSAALFPSDAGAAGDALTAWEAEFARQSFLNLCPLLHLAAVAVNEIILETTRNDKFIHIVDLGGIHHAHWVELLQGLATRRAAVRPCLRLTIVHEHKHFLGQAAQVLAAESDRHGVPLDLHIVESSVEALKLDALGVRSDHAVVIVSTLQLHRLVGAGILSTTAPPSPAAAAAASMITSPLPPANMSSKVDRLLRGFHLLSPRAIILTENEANHFVPSFTDRFASALPYYEQLFAAMEEAGAATVERKAAERYLLREEIKDVIACDHDGPRWARHETLGRWVVRMGAAGFALAPAITVVTAAGRVRAVAARLPGGGDERRYGVTEGGGWLILNREEKPMFCVSAWR	LCR1	5-20	AATAAATTTAAATTAA
				LCR2	32-52	MVPVPVASMATATAPAAVAAA
				LCR3	55-83	GGHGSSSASQNASGSGEGQGGSMSLSLQL
				LCR4	89-141	TPTAAVAVSVPPMAAAPMMAGPAAAAPAPAPPLATMAVAQNASLAAVASALAA
OsGRAS25	GRAS	116-484	MIALLMECAAAMSVGNLAGANGALLELSQMASPYAASCGERLVAYFARAMAARLVGSWVGVVAPMAPPPSCGAINAAFRALYNVAPFARLAYLACNQAILEAFHGKRLVHIVDLDVVPGGALQWLSLLPALAARPGGPPVIRVTGFGMSASVLHDTGNQLAGLARKLCMFFEFYAVAKRPGDADAVADMPGRRPGEAVAVHWLRHAMYDAAGDDGASMRLVRWLEPAAVTLVEQERAHGGGGGHGRFLDRFVSALHHYSAVFDAMGASRPDGEDASRHLAEHGVLGREIANVLAVGGPARSSGREGPGSWREVLARHGFAHAGGGGGGRAQLVAAACPGGLGYTVAGDHDGTVRLGWKGTPLYAVSAWT	LCR1	13-36	HHQYLYSSSSSNLPLQQPLLSHHH
				LCR2	98-111	ADVEQVAVEDEEEA
OsGRAS26	GRAS	36-423	IQQLLLHCAAALESNDVTLAQQAMWVLNNIASSQGDPSQRLTSWLLRALVARACRLCAAAPAGAAVEFLERGRAPPWGRAMSVTELADYVDLTPWHRFGFTASNAAILRAVAGASAVHVVDLSVTHCMQWPTLIDVLSKRPGGAPAIRITVPSVRPAVPPLLAVSSSELGARLAIFAKSKGVQLEFNVVESATTTSPKKTSTTLCQELASVLSDPPSLGLRDGEAVVVNCQSWLRHVAPDTRDLFLDTVRALNPCLLTVTDEDADLGSPSLASRMAGCFDFHWILLDALDMSAPKDSPRRLEQEAAVGRKIESVIGEEDGAERSEPGARLAERMSRKGFAGVVFDEEAAAEVRRLLSEHATGWGVKREDDMLVLTWKGHAAVFTGAWT	LCR	9-20	GGGVGAAAHGHG
OsGRAS28	GRAS	126-479	LVDDLLDAARLLDAGDSTSAREILARLNHRLPSLPSPPGHAHPPLLRAAALLRDALLPPTALPVSSTPLDVPLKLAAHKALADASPTVQFTTFTSTQAFLDALGSARRLHLLDFDVGFGAHWPPLMQELAHHWRRAAGPPPNLKVTALVSPGSSHPLELHLTNESLTRFAAELGIPFEFTALVFDPLSSASPPLGLSAAPDEAVAVHLTAGSGAFSPAPAHLRVVKELRPAVVVCVDHGCERGALNLLQSCAALLESLDAAGASPDVVSKVEQFVLRPRVERLAVGGGDKLPPPLQSMLASAGFAALQVSNAAEAQAECLLRRTASHGFHVEKRQAALALWWQRSELVSVSAWR	LCR	35-66	SSPSTSLGSCSSKPPEDPPPPIAADDDCDWDA
OsCIGR1	GRAS	201-571	VKQLLTRCAEALSEDRTEEFHKLVQEARGVVSINGEPIQRLGAYLLEGLVARHGNSGTNIYRALKCREPESKELLSYMRILYNICPYFKFGYMAANGAIAEALRTENNIHIIDFQIAQGTQWITLIQALAARPGGPPRVRITGIDDPVSEYARGEGLDIVGKMLKSMSEEFKIPLEFTPLSVYATQVTKEMLEIRPGEALSVNFTLQLHHTPDESVDVNNPRDGLLRMVKGLSPKVTTLVEQESHTNTTPFLMRFGETMEYYSAMFESIDANLPRDNKERISVEQHCLAKDIVNIIACEGKDRVERHELLGKWKSRLTMAGFRPYPLSSYVNSVIRKLLACYSDKYTLDEKDGAMLLGWRSRKLISASAWH			
OsGRAS32	GRAS	68-434	LLSLLLRCAEAVAMDQLPEARDLLPEIAELASPFGSSPERVAAYFGDALCARVLSSYLGAYSPLALRPLAAAQSRRISGAFQAYNALSPLVKFSHFTANQAIFQALDGEDRVHVIDLDIMQGLQWPGLFHILASRPTKPRSLRITGLGASLDVLEATGRRLADFAASLGLPFEFRPIEGKIGHVADAAALLGPRHHGEATVVHWMHHCLYDVTGSDAGTVRLLKSLRPKLITIVEQDLGHSGDFLGRFVEALHYYSALFDALGDGAGAAEEEAAERHAVERQLLGAEIRNIVAVGGPKRTGEVRVERWGDELRRAGFRPVTLAGSPAAQARLLLGMYPWKGYTLVEEDGCLKLGWKDLSLLTASSWE	LCR1	8-45	RAPGADAAAMKAKRAADDEEEGGERERARGKRLAAEGK
				LCR2	55-62	EEEEAAAE
				LCR3	437-449	DGDADADVAVAGD
OsCIGR2	GRAS	174-544	LKELLIACARAVEEKNSFAIDMMIPELRKIVSVSGEPLERLGAYMVEGLVARLASSGISIYKALKCKEPKSSDLLSYMHFLYEACPYFKFGYMSANGAIAEAVKGEDRIHIIDFHISQGAQWISLLQALAARPGGPPTVRITGIDDSVSAYARGGGLELVGRRLSHIASLCKVPFEFHPLAISGSKVEAAHLGVIPGEALAVNFTLELHHIPDESVSTANHRDRLLRMVKSLSPKVLTLVEMESNTNTAPFPQRFAETLDYYTAIFESIDLTLPRDDRERINMEQHCLAREIVNLIACEGEERAERYEPFGKWKARLTMAGFRPSPLSSLVNATIRTLLQSYSDNYKLAERDGALYLGWKSRPLVVSSAWH			
OsSHR1	GRAS	192-601	ASQLLLECARSVAARDSQRVQQLMWMLNELASPYGDVEQKLASYFLQGLFARLTASGPRTLRTLAAASDRNTSFDSTRRTALRFQELSPWSSFGHVAANGAILESFLEVAAAASSETQRFHILDLSNTFCTQWPTLLEALATRSADETPHLSITTVVSAAPSAPTAAVQRVMREIGQRMEKFARLMGVPFRFRAVHHSGDLAELDLDALDLREGGATTALAVNCVNSLRGVVPGRARRRDAFAASLRRLDPRVVTVVEEEADLVASDPDASSATEEGGDTEAAFLKVFGEGLRFFSAYMDSLEESFPKTSNERLALERGAGRAIVDLVSCPASESMERRETAASWARRMRSAGFSPVAFSEDVADDVRSLLRRYREGWSMREAGTDDSAAGAGVFLAWKEQPLVWASAWR	LCR1	11-40	QAASEQQQQQQQSASYNSRSTTSSGSRSSS
				LCR2	46-80	SYSYYHHSSNSGGGGGGGGGYYYGGQQPPPSQYYY
				LCR3	122-147	PPASSTPTGTAPTPPLSTSSTAAGAG
OsGRAS35	GRAS	76-471	MEQLLVHCANAIEANDATLTQQILWVLNNIAPADGDSNQRLTAAFLCALVSRASRTGACKAVTAAVADAVESAALHVHRFTAVELASFIDLTPWHRFGYTAANAAIVEAVEGFPVVHIVDLSTTHCMQIPTLIDMLAGRAEGPPILRLTVADVAPSAPPPALDMPYEELGAKLVNFARSRNMSMDFRVVPTSPADALTSLVDQLRVQQLVSDGGEALVVNCHMLLHTVPDETAGSVSLTTAQPPVSLRTMLLKSLRALDPTLVVVVDEDADFTAGDVVGRLRAAFNFLWIPYDAVDTFLPKGSEQRRWYEAEVGWKVENVLAQEGVERVERQEDRTRWGQRMRAAGFRAAAFGEEAAGEVKAMLNDHAAGWGMKREDDDLVLTWKGHNVVFASAWA	LCR1	9-25	PPPPPLHPNGHGLGLGL
				LCR2	34-54	GGGGARPWSSSSSTTTLGGSG
OsSCR1	GRAS	283-644	LLTLLLQCAESVNADNLDEAHRALLEIAELATPFGTSTQRVAAYFAEAMSARLVSSCLGLYAPLPNPSPAAARLHGRVAAAFQVFNGISPFVKFSHFTANQAIQEAFEREERVHIIDLDIMQGLQWPGLFHILASRPGGPPRVRLTGLGASMEALEATGKRLSDFADTLGLPFEFCPVADKAGNLDPEKLGVTRREAVAVHWLRHSLYDVTGSDSNTLWLIQRLAPKVVTMVEQDLSHSGSFLARFVEAIHYYSALFDSLDASYSEDSPERHVVEQQLLSREIRNVLAVGGPARTGDVKFGSWREKLAQSGFRVSSLAGSAAAQAVLLLGMFPSDGYTLIEENGALKLGWKDLCLLTASAWR	LCR1	3-55	SSSLLLFPSSSSSATHSSYSPSSSSHAITSLLPPLPSDHHLLLYLDHQEQHHL
				LCR2	86-101	AAAAPSSASAQLPALP
				LCR3	115-134	AAPAPPPPQQQVAAGEGGPP
				LCR4	148-157	ASSGAAVSVA
				LCR5	188-229	SDPAPPPPPPPSHPALLPPDATAPPPPPTSVAALPPPPPPQP
				LCR6	235-279	EPQCQEQEPNQPQSPKPPTAEETAAAAAAAKERKEEQRRKQRDEE

ΨOsGRAS8	GRAS	199-356	LRELLMSCAQAVASGNRRSAGELLEQIKRHSSPTGDATERLAHYFADGLEARLAGAASLERRLVASAEERASAMELLEAYQVFMAACCFKWVAFTFANMAILRAAEGRNRLHIVDYGGQYHGLQWPSLLQRLAEREGGPPEFRAVAAARWETVTAEDV	LCR1	29-40	PAAPPSEAAAAA
	GRAS	339-542	EFRAVAAARWETVTAEDVVGVDPDDEAAVVVNDVLSLGTLMDESGVFDDPSPRDTVLGSIRDMRPAVFVQAVVNGAHGAPFFPTRFREALFFFSALFDMLGATTPEEGSHLRVVLERDVLRRAAVGVIAGEGAERVERPETYRRWQARNRRAGLRQAAVEGDVVEAVRRRVRRRHHEEFVIEEDAGWLLQGWKGRILYAHSAWV	LCR2	108-122	GSGNGRGRKGSKHGG
				LCR3	140-147	EEEEDDDD
				LCR4	177-193	AEKKCGKAARRRRRQAK
OsGRAS39	GRAS	67-365	RDVLVVHIVDLSCSAAHPWQWPKLLDDFHGRPGGAPELYLTVLHDDNDFLADMQSLLSKKAESLGVSFHFISVIGRLETLDFSNLRSTFQIKFGVAVAISCALQMHRLLLVDDNLSSTSIAQLQKMANFTQPKQMASSVCSPASTLNYLQTPSPRTPKLLARLLSAIRALKPNIMLIMEQDADHNTLLFRDRFNEVLNYYAALFDCFHAVAAANPGRTDERLRVDRMILREEIKNILVCEGVHRHERHERLDQWAMHMEESGFHNVQLSFSAIREAYVWQLKVQADNLRLCCTDRGMFQ	LCR	370-391	SSATSSPASSVYSPSPSPSNGS
	GRAS	405-843	LIGLLYQCAAEVSAGSFDRANLCLEHITQLASLDAPHALQRLAAVFADALARKLLNLILGLSRALLSSANSADAHLVPVARRHMFDVLPFLKLAYLTTNHAILEAMEGERFVHVVDFSGPAANPVQWIALFHAFRGRREGPPHLRITAVHDSKEFLANMAAVLSKEAEAFDIAFQFNAVEAKLDEMDFDALRHDLGVRSGEALAVSVVLQLHRLLAVDDGRRHAAAGCLTPVQIIARSSPRSFGELLERELNTRLQLSPDASVVSSLSPHSPAAATAAHPTTSTPKLGSFLSAVRSLSPKIMVMTEQEANHNGGAFQERFDEALNYYASLFDCLQRSAAAAAERARVERVLLGEEIRGVVACEGAERVERHERARQWAARMEAAGMERVGLSYSGAMEARKLLQSCGWAGPYEVRHDAGGHGFFFCWHKRPLYAVTAWR			
OsGRAS41	GRAS	4-339	LSDLLLAGAEAVEAGDSILASVAFSRLDDFLSGIPENGAASSFDRLAYHFDQGLRSRMSSASTGCYQPEPLPSGNMLVHQIIQELSPFVKFAHFTTNQAILDAIIGDMDVHVVDLNIGEGIQWSSLMSDLARCGGKSFRLTAITTYADCHASTHDTVVRLLSEFADSLELPFQYNSICVHNEDELHAFFEDCKGSVIVSCDTTSMYYKSLSTLQSLLLVCVKKLQPKLVVTIEEDLVRIGRGVSPSSASFVEFFFEALHHFTTVFESMASCFIGSSYEPCLRLVEMELLGPRIQDFVVKYGSVRVEANASEVLEGFMACELSACNIAQARMLVGLFNRVFGVVFKKISLLMVY			
	Transmembrane region	354-376	VIWSSLAAGCGSHGIVVLAFYAA			
ΨOsGRAS9	GRAS	1-129	MSHLENTLEARLAGTGSQMYQSLVAKRTSTVDFLKAYKLFTAACCVKKTIYNAVAGKRKLHIVDYGLSYGFQWPALFFLLGTREGGPPEVRMTGIDVPQPGFRPADQIEETGRRLSICARAPVRCAIQV	LCR	216-254	SRSAPSSSPPRPSLHLHLHLRRRPPPSSSRHAADDAALH
OsGRAS43	RPT1	94-113	ASPRRDFMACSPKRDYMVTT	LCR1	2-13	AGGGAKLQQQQA
	RPT1	114-134	SSPKRDYMVTSSPKRDYMVSS	LCR2	165-177	HGGGGGGGHHLHH
	GRAS	401-771	LVHLLLACADLVSKGDHPAALRHLHLLRRVASPLGDSMQRVASHFADALAARLSLLSSPTSASPSPRAAAAAAPYPFPPSPETLKVYQILYQACPYIKFAHFTANQAIFEAFHGEDRVHVVDLDILQGYQWPAFLQALAARPGGPPTLRLTGVGHPPAAVRETGRHLASLAASLRVPFEFHAAAADRLERLRPAALHRRVGEALAVNAVNRLHRVPSSHLPPLLSMIRDQAPKIITLVEQEAAHNGPYFLGRFLEALHYYSAIFDSLDATFPAESTARMKVEQCLLAPEIRNVVACEGAERVARHERLERWRRLMEGRGFEAVPLSAAAVGQSQVLLGLYGAGDGYRLTEDSGCLLLGWQDRAIIAASAWR	LCR3	183-201	GGGMEGGGGGHGAQPQYGG
				LCR4	223-230	GGGSGGGG
				LCR5	280-288	GGVGGGGGG
				LCR6	293-306	SGASVSVVTAPASS
				LCR7	326-341	GGGDEAVAAAMAVAGE
				LCR8	355-364	GGGGEFGGEG
OsGRAS44	GRAS	303-683	LHTLLIHCAQAVATSDRRSATELLKQIKQNSSARGDATQRLACCFAEGLEARLAGTGSQVYKSLVAKCTSTVDFLKAYKLFAAACCIKKVSFIFSNKTILDAVAGKRKLHIVDYGLSYGFQWPGLFKCLSEREGGPPEVRITGIDFPQPGFRPADQIEETGRRLSNCARQFGVPFRFQAIAAKWETVRREDLHLDREEEEEEEEEVLVVNCLHFLNALQDESVVVDSPSPRDMVLNNIRDMRPHVFVQCVVNGAYGAPFFLTRFRETLFFYSSQFDMLDATIPRDNDERLLIERDILGRWALNVIACEGADRVDRPETYKQWLVRNHRAGLTQLPLQPQVVELVRDKVKKLYHKDFVIDVDHNWLLQGWKGRILYAMSTWV	LCR1	14-27	LEPFSPSLFLDLPP
				LCR2	82-111	SDDTTTNSSDDSASATTNNTTNSAAAANAS
				LCR3	209-218	GRSGGSGRGR
				LCR4	279-299	AEKKARNGGGAGRRAARAKAA
OsGRAS47	GRAS	215-586	LRMLLIQCAQAMATDNQQSAGELLKKIKQHALATGDAMQRVAHYFAKGLEARLAGSGKHLYQNHVRMSLVEYLKVYKLYMAACCFKKVALMFAAMTIMQAVQGKKRLHIVDYGIRCGLHWPDLFRRLGSREDGPPEVRITIVDIPQPGFRPFQRIEAAGHCLSSCANEFRVPFRFQAVVAAKWETVGAEDLHIEPDEVLVVNDLWSFSALMDESIFCDGPNPRDVALRNISKMQPDVFIQGIINGGYGASFLSRFRGALLYYSALFDMLDATTPRESGLRLALEQNVLGPYALNAIACEGADLVERPEKYRQWQARNHRAGMQQLKLRPDIVDTIREEVNKYHHKDFLLGEDGQWLLQGWMGRVLFAHSAWV	LCR	201-211	KKKGKKGSSSK
ΨOsGRAS10	RPT1	61-73	FLDMMVIQESANE	LCR1	22-30	SSSSLLLWS
	RPT1	117-129	FLEMMAIQESAND	LCR2	47-60	DADHSHDQIHQDHQ
	GRAS	173-308	AGDLLLAGAMAVDAGDAVHASAIMSRLDDLLADIAGRRSCEATSPVDHLAYYFARGLKLRISGAATPASSPPPPAANWSSPAYRMLQELTPFVKFAHFTANQAILEATADDLDVHVVDFNVGEGVQWSSLMLKLLL			
	GRAS	305-460	KLLLLGTITILQPKLVILIEDELSRISKNPPSPSLAAPPPFPEFFSDAVAHFTAVMESTASCLVSYDDEAWLSLRRVGEEVVGPRVEDAVGRYGSLAGGAQMMEGLRAREVSGFSVAQGKMLAGLFGGGFGVVHQEKGRLALCWKSRPLISVSLWC			
OsGRAS53	RPT1	190-207	FLKGMEEANKFLPTENKL	LCR1	53-65	PPSPPPPTTTATT
	RPT1	219-236	YLRGLEEAKRFLPSDDKL	LCR2	116-135	LSDPSSNSRSSNSDDPRLSP
	GRAS	358-730	LRTLLIHCAQAVATDDRRSATELLKQIKQHAKPTGDATQRLAHCFAEGLQARIAGTGSLVHQSLVAKRTSAVDILQAYQLYMAAICFKKVSFIFSNQTIYNASLGKKKIHIVDYGIQYGFQWPCFLRRISQREGGPPEVRMTGIDLPQPGFRPTERIEETGHRLSKYAQEFGVPFKYNAIAAVKMESVRKEDLNIDPDEVLIVNCQYQFKNLMDESVVIDSPRDIVLSNIRKMQPHVFIHAIVNGSFSAPFFVTRFREALFFYSALFDVLDATTPRESEQRLLIEQNIFGRAALNVIACEGIDRVERPETYKQWQVRNQRAGFKQLPLNPEIVQVVRNKVKDCYHKDFVIDIDHQWLLQGWKGRILYAISTWT	LCR3	150-162	AAATATAVAAAAV
				LCR4	241-260	AAAAAPVVSVKKEAVDVVVA
				LCR5	337-356	GGKGGNGKVKGGRRGGRDVV

Supplementary Table S4: List of GRAS genes and their expression patterns under NaCl and ABA treatments

Gene name	Locus number	Regulation (up/down)	Type of Response	Maximum fold change	Regulation (up/down)	Type of Response	Maximum fold change
		Root			Shoot		
**NaCl**							
OsGRAS1	LOC_Os01g45860	DOWN	-	-	-	-	-
OsGRAS2	LOC_Os01g62460	UP	E(2.9)	3.3	-	-	-
OsGRAS3	LOC_Os01g65900	DOWN	-	-	-	-	-
OsGRAS5	LOC_Os01g71970	UP	IE (5.5)	6.1	-	-	-
OsGRAS7	LOC_Os02g10360	DOWN	-		DOWN	-	-
ΨOsGRAS2	LOC_Os02g21685	UP	E(2.6)	18.8	DOWN	-	-
OsGRAS8	LOC_Os02g44360	UP	IE(5.6)	12	DOWN	-	-
OsGRAS10	LOC_Os02g45760	UP	IE(12.4)	12.4	DOWN	-	-
OsGRAS11	LOC_Os03g09280	UP	IE(3.8)	4.8	DOWN	-	-
OsGRAS12	LOC_Os03g15680	UP	IE(9.5)	9.5	UP	E(2.1)	6.8
OsSHR2	LOC_Os03g31880	UP	IE(3.6)	4.3	DOWN	-	-
ΨOsGRAS3	LOC_Os03g37900	UP	IE(3.7)	3.7	DOWN	-	-
ΨOsGRAS4	LOC_Os03g40080	UP	IE(3.0)	16.2	DOWN	-	-
OsGRAS15	LOC_Os03g48450	UP	IE(3.8)	3.8	DOWN	-	-
OsSLR1	LOC_Os03g49990	UP	IE(3.3)	11	DOWN	-	-
OsGRAS18	LOC_Os03g51330	-	-	-	DOWN	-	-
OsGRAS19	LOC_Os04g35250	UP	IE(4.2)	6.4	UP	IE(2.1)	2.1
ΨOsGRAS5	LOC_Os04g37440	UP	IE(2.2)	2.2	UP	IE(7.7)	25.2
OsGRAS20	LOC_Os04g46860	DOWN	-	-	DOWN	-	-
OsGRAS22	LOC_Os04g50060	UP	IE(3.2)	4.6	DOWN	-	-
OsGRAS23	LOC_Os05g31380	DOWN			DOWN	-	-
OsGRAS24	LOC_Os05g31420	UP	IE(4.0)	8.6	UP	L(4.6)	4.6
OsGRAS25	LOC_Os05g40710	UP	E(4.5)	4.5	UP	E(4.8)	4.8
OsGRAS26	LOC_Os05g42130	UP	IE(2.6)	2.6	DOWN	-	-
OsGRAS28	LOC_Os06g01620	DOWN	-	-	DOWN	-	-
OsCIGR1	LOC_Os07g36170	UP	IE(3.2)	3.2	DOWN	-	-
OsGRAS32	LOC_Os07g38030	DOWN	-	-	DOWN	-	-
OsCIGR2	LOC_Os07g39470	DOWN	-	-	DOWN	-	-
OsSHR1	LOC_Os07g39820	UP	IE(7.3)	11.1	DOWN	-	-
OsGRAS35	LOC_Os07g40020	UP	E(6.8)	17.4	DOWN	-	-
OsSCR1	LOC_Os11g03110	UP	E(3.8)	5.7	DOWN	-	-
ΨOsGRAS8	LOC_Os11g04400	DOWN			DOWN	-	-
OsGRAS39	LOC_Os11g04570	UP	IE(3.6)	65.3	DOWN	-	-
OsGRAS41	LOC_Os11g06180	UP	IE(2.4)	4.1	DOWN	-	-
ΨOsGRAS9	LOC_Os11g11600	UP	L(8.0)	8	DOWN	-	-
OsGRAS43	LOC_Os11g31100	UP	IE(3.5)	3.5	DOWN	-	-
OsGRAS44	LOC_Os11g47870	UP	IE(2.9)	5.8	DOWN	-	-
OsGRAS47	LOC_Os11g47910	-	-	-	DOWN	-	-
ΨOsGRAS10	LOC_Os12g06540	DOWN	-	-	DOWN	-	-
OsGRAS53	LOC_Os12g38490	DOWN	-	-	DOWN	-	-

Gene name	Locus number	Regulation (up/down)	Type of Response	Maximum fold change	Regulation (up/down)	Type of Response	Maximum fold change
		Root			Shoot		
**ABA**							
OsGRAS1	LOC_Os01g45860	-	-	-	DOWN	-	-
OsGRAS2	LOC_Os01g62460	UP	IE(3.3)	5.6	DOWN	-	-
OsGRAS3	LOC_Os01g65900	UP	L(3.5)	3.5	DOWN	-	-
OsGRAS5	LOC_Os01g71970	UP	IE(6.2)	12.2	DOWN	-	-
OsGRAS7	LOC_Os02g10360	DOWN	-	-	DOWN	-	-
ΨOsGRAS2	LOC_Os02g21685	UP	IE(2.8)	32.7	UP	L(3.5)	3.5
OsGRAS8	LOC_Os02g44360	UP	IE(5.5)	27.9	DOWN	-	-
OsGRAS10	LOC_Os02g45760	UP	IE(2.4)	16.7	DOWN	-	-
OsGRAS11	LOC_Os03g09280	UP	L(4.1)	4.1	DOWN	-	-
OsGRAS12	LOC_Os03g15680	UP	IE(8.2)	8.2	UP	L(3.8)	3.8
OsSHR2	LOC_Os03g31880	UP	IE(4.5)	14.3	DOWN	-	-
ΨOsGRAS3	LOC_Os03g37900	UP	IE(2.8)	2.8	DOWN	-	-
ΨOsGRAS4	LOC_Os03g40080	UP	IE(2.1)	2.1	DOWN	-	-
OsGRAS15	LOC_Os03g48450	UP	IE(2.9)	14.6	DOWN	-	-
OsSLR1	LOC_Os03g49990	UP	IE(7.9)	30.2	DOWN	-	-
OsGRAS18	LOC_Os03g51330	UP	IE(2.8)	3.4	DOWN	-	-
OsGRAS19	LOC_Os04g35250	UP	L(3.5)	11	DOWN	-	-
ΨOsGRAS5	LOC_Os04g37440	UP	IE(2.9)	2.9	UP	IE(8.2)	31.6
OsGRAS20	LOC_Os04g46860	UP	IE(2.9)	4.1	DOWN	-	-
OsGRAS22	LOC_Os04g50060	UP	E(5.5)	14.2	DOWN	-	-
OsGRAS23	LOC_Os05g31380	DOWN	-	-	DOWN	-	-
OsGRAS24	LOC_Os05g31420	UP	IE(15.3)	15.3	UP	L(6.9)	6.9
OsGRAS25	LOC_Os05g40710	UP	IE(6.2)	14.2	UP	IE(3.0)	10.5
OsGRAS26	LOC_Os05g42130	UP	L(2.4)	2.4	DOWN	-	-
OsGRAS28	LOC_Os06g01620	DOWN	-	-	DOWN	-	-
OsCIGR1	LOC_Os07g36170	UP	IE(7.3)		DOWN	-	-
OsGRAS32	LOC_Os07g38030	UP	IE(2.09)		DOWN	-	-
OsCIGR2	LOC_Os07g39470	DOWN			DOWN	-	-
OsSHR1	LOC_Os07g39820	UP	IE(14.1)	66.3	DOWN	-	-
OsGRAS35	LOC_Os07g40020	UP	IE(15.9)	15.9	DOWN	-	-
OsSCR1	LOC_Os11g03110	UP	IE(6.7)	34.3	UP	L(2.4)	2.4
ΨOsGRAS8	LOC_Os11g04400	DOWN	-	-	DOWN	-	-
OsGRAS39	LOC_Os11g04570	UP	IE(12)	101.4	DOWN	-	-
OsGRAS41	LOC_Os11g06180	UP	IE(5)	21.3	DOWN	-	-
ΨOsGRAS9	LOC_Os11g11600	UP	L(2.7)	2.7	DOWN	-	-
OsGRAS43	LOC_Os11g31100	UP	IE(2.07)	7.3	DOWN	-	-
OsGRAS44	LOC_Os11g47870	UP	IE(19.2)	19.2	DOWN	-	-
OsGRAS47	LOC_Os11g47910		-	-	DOWN	-	-
ΨOsGRAS10	LOC_Os12g06540	DOWN	-	-	DOWN	-	-
OsGRAS53	LOC_Os12g38490	DOWN	-	-	DOWN	-	-
